# Homo- and Hetero-Dimers of CAD Enzymes Regulate Lignification and Abiotic Stress Response in Moso Bamboo

**DOI:** 10.3390/ijms222312917

**Published:** 2021-11-29

**Authors:** Naresh Vasupalli, Dan Hou, Rahul Mohan Singh, Hantian Wei, Long-Hai Zou, Kim Yrjälä, Aimin Wu, Xinchun Lin

**Affiliations:** 1 State Key Laboratory of Subtropical Silviculture, Zhejiang A & F University, Hangzhou 311300, China; naribiotech@gmail.com (N.V.); 20184007@zafu.edu.cn (D.H.); 2018102561002@stu.zafu.edu.cn (H.W.); zoulonghai@zafu.edu.cn (L.-H.Z.); kim.yrjala@helsinki.fi (K.Y.); 2 Shanghai Center for Plant Stress Biology, CAS Center for Excellence in Molecular Plant Sciences, Chinese Academy of Sciences, Shanghai 200032, China; rahul.mohan@psc.ac.cn; 3 Department of Forest Sciences, University of Helsinki, 00014 Helsinki, Finland; 4 Guangdong Key Laboratory for Innovative Development and Utilisation of Forest Plant Germplasm, College of Forestry and Landscape Architecture, South China Agricultural University, Guangzhou 510642, China; wuaimin@scau.edu.cn; 5 State Key Laboratory for Conservation and Utilisation of Subtropical Agro-Bioresources, South China Agricultural University, Guangzhou 510642, China

**Keywords:** Moso bamboo, lignin, CAD, yeast two-hybrid, BiFC, pull-down

## Abstract

Lignin biosynthesis enzymes form complexes for metabolic channelling during lignification and these enzymes also play an essential role in biotic and abiotic stress response. Cinnamyl alcohol dehydrogenase (CAD) is a vital enzyme that catalyses the reduction of aldehydes to alcohols, which is the final step in the lignin biosynthesis pathway. In the present study, we identified 49 CAD enzymes in five Bambusoideae species and analysed their phylogenetic relationships and conserved domains. Expression analysis of Moso bamboo *PheCAD* genes in several developmental tissues and stages revealed that among the *PheCAD* genes, *PheCAD2* has the highest expression level and is expressed in many tissues and *PheCAD1*, *PheCAD6*, *PheCAD8* and *PheCAD12* were also expressed in most of the tissues studied. Co-expression analysis identified that the *PheCAD2* positively correlates with most lignin biosynthesis enzymes, indicating that *PheCAD2* might be the key enzyme involved in lignin biosynthesis. Further, more than 35% of the co-expressed genes with *PheCADs* were involved in biotic or abiotic stress responses. Abiotic stress transcriptomic data (SA, ABA, drought, and salt) analysis identified that *PheCAD2*, *PheCAD3* and *PheCAD5* genes were highly upregulated, confirming their involvement in abiotic stress response. Through yeast two-hybrid analysis, we found that *PheCAD1*, *PheCAD2* and *PheCAD8* form homo-dimers. Interestingly, BiFC and pull-down experiments identified that these enzymes form both homo- and hetero- dimers. These data suggest that *PheCAD* genes are involved in abiotic stress response and *PheCAD2* might be a key lignin biosynthesis pathway enzyme. Moreover, this is the first report to show that three PheCAD enzymes form complexes and that the formation of PheCAD homo- and hetero- dimers might be tissue specific.

## 1. Introduction

Lignin is the major component of the secondary cell wall and is the most abundant aromatic compound produced by plants. Lignin is more hydrophobic than cellulose, thereby playing a critical role in water transport in the xylem vessels [1,2]. Due to its rigidity, lignin also plays a crucial role in plant defence [3]. Thus, abiotic and biotic stresses trigger the induction of the lignin polymer synthesis [3]. Lignin is derived from the polymerisation of three phenylpropanoid monomers *p*-coumaryl, coniferyl and sinapyl alcohols also called *p*-hydroxyphenyl (H), guaiacyl (G) and syringyl (S) monolignols, respectively [4]. A sequence of eleven phenylpropanoid pathway enzyme families is involved in the biosynthesis of monolignols, starting from the deamination of phenylalanine to monolignol precursors in the cytoplasm (Figure S1) [4]. The composition of monolignols is highly diverse across plant taxa, cell types, developmental stages and cell wall layers [5]. For example, lignin is polymerised from S and G monolignols in angiosperms, whereas in gymnosperms, lignin is composed of purely G lignin [5].

Cinnamyl alcohol dehydrogenase (CAD) is the last enzyme in the lignin biosynthesis pathway, and it reduces sinapaldehyde, coniferaldehyde and *p*-coumaraldehyde to sinapyl, coniferyl and *p*-coumaryl alcohols, respectively [6]. CAD enzymes contain catalytic and structural Zn-binding sites and the NADP(H) binding sites [7]. Due to the economic importance and involvement in various biological roles, CAD enzyme has been well studied in several plant species. Earlier studies showed a significant reduction in the lignin content in the natural pine and maize CAD mutants [8,9]. Further, downregulation of *CAD* genes expression in tobacco, pine and poplar results in the incorporation of sinapaldehyde and coniferaldehyde in place of sinapyl and coniferyl alcohols in the lignin polymer [8,10,11,12]. The incorporation of aldehyde molecules imparts the reddish-brown colour to the stems of both natural and induced CAD mutants in *Zea mays*, *Oryza sativa*, pine and poplar [8,9,13]. Similarly, in Arabidopsis double mutant (*cad-c cad-d*), coniferyl and sinapyl aldehydes were incorporated in place of coniferyl and sinapyl alcohols resulting in a limp floral stem [14]. Moreover, a significant reduction in the stem lignin content was also observed in these natural or induced mutants [8,9,14].

*AtCAD-D* gene acts as a crucial component for resistance to *Pseudomonas syringae* pv. *Tomato* infection [15]. Expression of *CAD1* gene in *Ipomoea batatas* is highly induced by cold, reactive oxygen species and wounding [16]. Overexpression of *I. batatas CAD1* and *Sedum alfredii CAD* gene in Arabidopsis increases oxidative stress tolerance and cadmium stress tolerance, respectively [17,18]. *Ginkgo biloba CAD1* gene expression is increased after SA, UV, ethephon and wounding treatment [19]. Similarly, *CAD1* and *CAD2* genes of *Plagiochasma appendiculatum* are induced by MeJA treatment [20]. Deng et al. (2013) [21] identified the *CsCAD3* gene was highly upregulated after wounding and *Ectropis oblique* attack. Further, *CsCAD1* and *CsCAD2* had elevated expression after MeJA and SA treatment. Likewise, the *Cucumis melo CmCAD2* gene becomes strongly upregulated after salt, drought and wounding treatments [22].

Bamboo is the fastest-growing plant (7.5–100 cm per day) and is used for human food, animal diet, timber, textile, musical instruments, fuel and paper production [23,24,25]. The industry related to bamboo was estimated to be USD 72.1 billion in 2019 (www.globenewswire.com, accessed on 1 November 2021). The fast growth of bamboo forests promotes more than 30% more high-carbon sequestration than the tropical mountain rainforest and fast-growing Chinese fir, *Cunninghamia lanceolata* [26,27]. Moso bamboo (*Phyllostachys edulis*) is the most crucial bamboo in China, covering around 3 million ha, and is the main species for edible shoot, timber and paper production. Moso bamboo is a tetraploid woody plant containing 48 chromosomes (2n = 4X = 48) [28]. Peng et al. (2013) [29] published the first scaffold draft genome for Moso bamboo. Zhao et al. (2018) [30] recently released the chromosome-level reference genome and also provided the transcriptomic data for multiple developmental tissues belonging to different heights of Moso bamboo. Recently, the draft genome sequences of diploid herbaceous bamboo (*Raddia guianensis* and *Olyra latifolia*), tetraploid neotropical woody bamboo (*Guadua angustifolia*) and hexaploid palaeotropical woody bamboo (*Bonia amplexicaulis*) were published [31].

Lignin biosynthesis enzymes interact with each other and forms complexes for metabolic channelling [32]. Recently Yan et al. (2019) [33] identified that *Populus trichocarpa* PtrCAD1 forms homo-dimers through BiFC experiments, but it needs more studies in different plant species to characterise the CAD enzyme complexes. Further, the *CAD* genes response to abiotic or biotic stress data is also lacking in Moso bamboo. Therefore, the availability of whole-genome or draft genome sequences of different bamboo species, different kinds of Moso bamboo tissues and developmental stages transcriptomic data and our laboratory’s unpublished abiotic stress treatment transcriptomic data provided an opportunity to study the *CAD* gene family in Bambusoideae. In this study, we identified *CAD* genes belonging to five bamboo species and their phylogenetic relationship and conserved domains. Further, we analysed the *PheCAD* genes expression in different Moso bamboo tissues and identified the most expressed *PheCAD* genes, *PheCAD1*, *PheCAD2* and *PheCAD8*, forms both homo- and hetero- dimers in planta. Then we analysed the response of the *PheCAD* genes to abiotic stress and the co-expression network and identified the lignin biosynthesis genes co-expressed with *PheCAD* genes.

## 2. Results

### 2.1. Identification of Bambusoideae CAD Enzymes

Our yeast two-hybrid (Y2H) one-to-one interaction experiments to identify the enzyme–enzyme interactions of lignin biosynthesis enzymes identified the *PheCAD2* enzyme forms homo-dimers in Moso bamboo. To characterise the Moso bamboo CAD enzymes, we identified them from a *P. edulis* genome database using the rice CAD protein sequences (downloaded from the Oryzabase) as a reference. The enzymes containing the catalytic Zn-binding site “GHE(X)2G(X)5V”, structural Zn-binding site “GD(X)10C(X)2C(X)7C” and the NADP(H) binding site “G(X)3G(X)2GLGG(X)GH(X)2VK(X)2K(X)2G-(X)VTV(X)S(X)S(X)2K” were considered as CAD enzymes [6]. A total of 14 PheCAD enzymes were identified in the Moso bamboo genome database (Table S1). Further, to study the phylogenesis and domains, we also isolated the CAD enzymes from available bamboo draft genome sequences *R. guianensis* (2n), *O. latifolia* (2n), *G. angustifolia* (4n), and *B. amplexicaulis* (6n) [31]. A total of 11 RguCAD, seven OlaCAD, eight GanCAD and 16 BamCAD enzymes were identified, respectively (Table S1). Most of the identified CAD enzymes contain intact catalytic and structural Zn-binding sites and the NADP(H) binding sites with minor mutations. Unlike other Bambusoideae species, six of the 11 RguCAD enzymes (RguCAD6-11) have mutations in the NADP(H) binding site (File S1). Further, in angiosperms, polyploidy is considered a vital mechanism for speciation and family expansion [34]. However, the number of CAD enzymes in Bambusoideae is poorly correlated with the polyploidisation, which may be due to the lower sequence and assembly quality of the draft genomes [31].

### 2.2. Evolution of Bambusoideae CAD Enzymes

**Figure 1:**
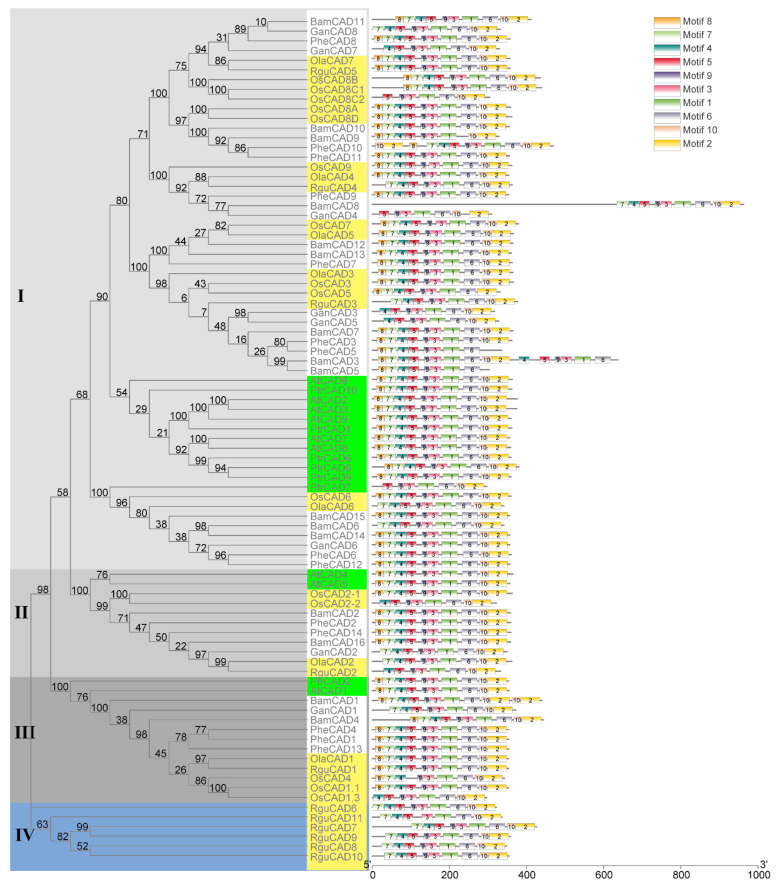
The phylogenetic relationship among *CAD* genes of Bambusoideae. The phylogenetic tree was constructed using CAD protein sequences of *P. trichocarpa* (Ptr), *Arabidopsis thaliana* (At), *Oryza sativa* (Os), *O. latifolia* (Ola), *P. edulis* (Phe), *G. angustifolia* (Gan), *R. guianensis* (Rgu) and *B. amplexicaulis* (Bam). Clade I, II and III are indicated in different ash shades and Clade IV in blue. The dicot plant *CAD* genes were indicated in the green boxes and the diploid rice and bamboo species are shown in light yellow boxes. The conserved motifs (1–10) are indicated in ten different colour boxes.

To understand the evolution of CAD enzymes, a phylogenetic tree containing 87 CAD enzymes (AtCADs, BamCADs, GanCADs, OlaCADs, OsCADs, PheCADs, PtrCADs and RguCADs) was constructed (Figure 1). The aligned amino acid sequences of CAD enzymes were used to build the phylogenetic tree using both a maximum likelihood and neighbor-joining method with 1000 bootstrap replicates [35]. The phylogenetic tree is divided into Clade I, Clade II, Clade III and Clade IV. Clade I contains more CAD enzymes than other Clades. The evolutionary topologies of the subclades are divided between monocot and dicot CAD enzymes. The OsCAD and diploid herbaceous bamboo OlaCAD, RguCAD enzymes are separated in the subclades compared to tetraploid and other woody bamboo CAD enzymes. Interestingly, the RguCAD6-11 enzymes which have mutations in the NADP(H) binding site are entirely separated in the phylogenetic tree (Figure 1 and Figure S2). These results support that CAD enzymes were present before the divergence of dicot and monocot plants, and also, they evolved separately in the Bambusoideae after polyploidisation.

### 2.3. Identification of Conserved Domains of the CAD Enzymes

To better understand the protein structures of CAD enzymes, we analysed the conserved motifs through MEME software. A total of ten conserved motifs were identified in the CAD enzymes in both monocot and dicot plants analysed in this study. Except for some, all ten motifs were present in the same order for most CAD enzymes (Figure 1). Interestingly, the starting three motifs 8, 7 and 4 are absent in the OsCAD8C2, GanCAD4 and PtrCAD7. The first motif, 8, is missing in 14 CAD enzymes and the starting two motifs 8 and 7 in six CAD enzymes. Further, the last two motifs 10 and 2, are absent in the BamCAD5 and PheCAD5 and duplicated in the *PheCAD10* and BamCAD1, whereas motif 6 and 5 are lacking in the BamCAD9 and OsCAD4. Moreover, motifs 4, 5, 9, 3, 1 and 6 are duplicated in the BamCAD3 enzyme (Figure 1 and Figure S2).

### 2.4. Cis-Acting Elements Responsive to Abiotic Stress

**Figure 2:**
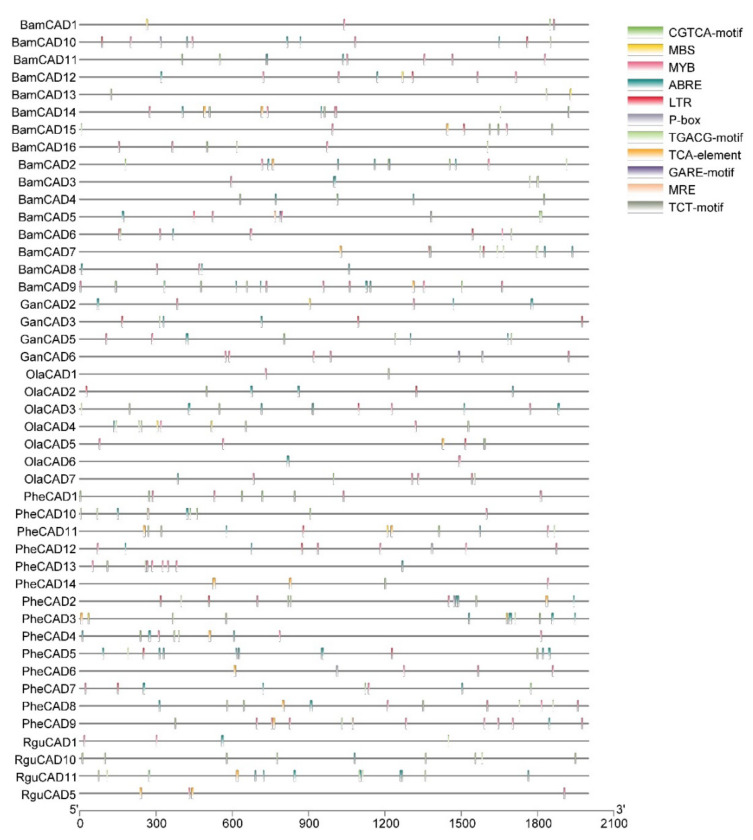
The conserved *cis*-elements analysis of *CAD* genes in the promoter regions of Bambusoideae (ABRE, CGTCA-motif, GARE-motif, LTR, MBS, MRE, MYB, P-box, TCA-element, TCT-motif and TGACG-motif).

*Cis*-acting elements trigger the genes involved in abiotic stress. Therefore, studying *cis*-acting elements in the promotor regions of *CAD* genes will help to understand their role in abiotic stress response. Hence, we analysed the 2kb upstream region to the *PheCAD*, *BamCAD*, *GanCAD*, *OlaCAD* and *RguCAD* genes. Due to the lack of proper genome assembly, we were unable to retrieve all the upstream 2kb regions of *GanCAD* and *RguCAD* genes. Further, among the *cis*-acting elements analysed we focused on ABA, SA and GA-responsive and other important *cis*-elements such as ABRE, CGTCA-motif, GARE-motif, LTR, MBS, MRE, MYB, P-box, TCA-element, TCT-motif and TGACG-motif (Figure 2 and Table S2).

### 2.5. Expression of Moso Bamboo CAD Genes during Plant Development

**Figure 3:**
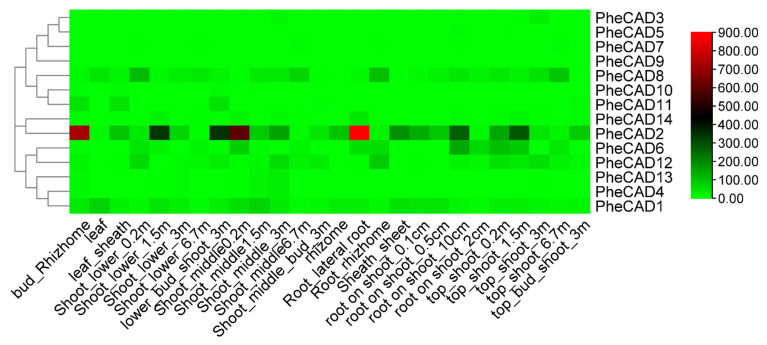
Expression heatmap of *PheCAD* genes in 26 different kinds of tissues and stages of bamboo development. The log2 expression values are depicted in colour shades from red (highest) to green (lowest).

We analysed the transcriptomic data of twenty-six bamboo tissues published by Zhao et al., (2018) [30] and used the FPKM (Fragments Per Kilobase of transcript per Million mapped reads) values of 14 *PheCAD* genes (Table S3). A heatmap was constructed using the FPKM values to identify the most-expressed genes (Figure 3). The five Moso bamboo *CAD* genes *PheCAD1*, *PheCAD2*, *PheCAD6*, *PheCAD8*, and *PheCAD12* are expressed in most of the analysed tissues. In these, the *PheCAD2* (PH02Gene39617) gene is highly expressed but not in the leaf and 6.7 m height shoot. At the same time, *PheCAD8* (PH02Gene13789) is also expressed in most tissues, including leaf and shoot 6.7 m. Further, *PheCAD1* (PH02Gene46268) is also expressed in different kinds of tissues, including leaves.

### 2.6. Co-Expression Analysis of PheCAD Genes in Moso Bamboo

**Figure 4:**
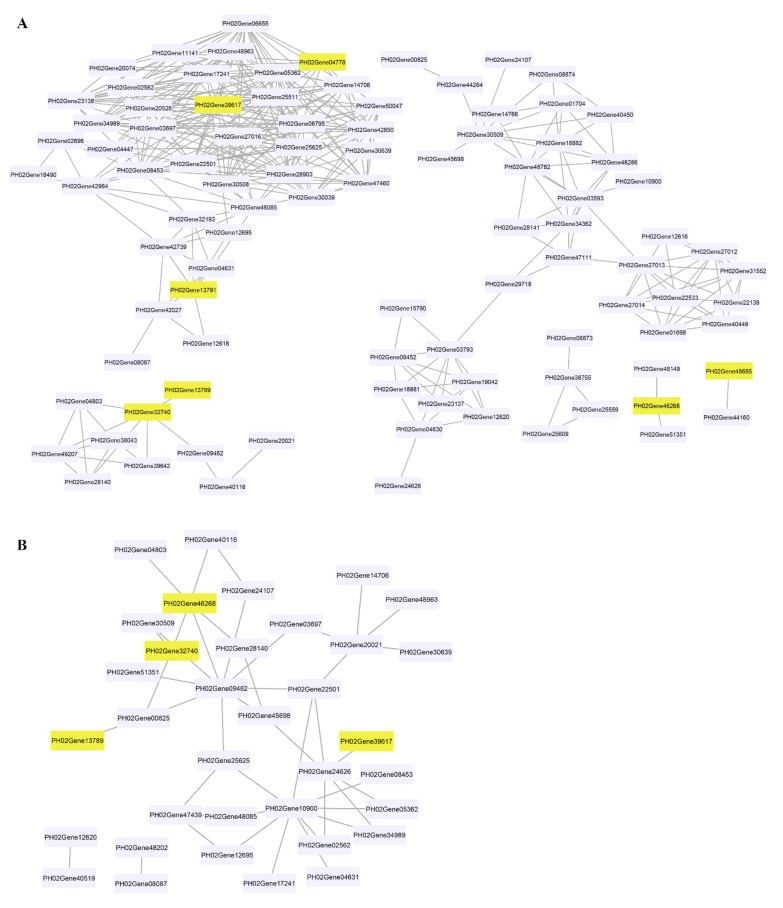
Co-expression network of *PheCAD* genes with lignin biosynthesis pathway genes. (**A**) Positive correlation of *PheCAD* genes with lignin biosynthesis pathway genes; (**B**) negative correlation of *PheCAD* genes with lignin biosynthesis pathway genes. Pink-coloured boxes indicate the *PheCAD* genes and violet boxes indicate the other lignin biosynthesis genes.

We analysed the gene co-expression network for lignin biosynthesis genes to identify the lignin biosynthesis genes co-expressed with *PheCAD* genes. A total of 160 lignin biosynthesis genes’ FPKM values in 26 different tissues were used for co-expression analysis (Table S3). The Pearson correlation coefficient (PCC) was calculated between two genes as the correlation coefficient to measure the co-expression relationship. The highest PCC values > 0.7 and lowest PCC values < −0.5 were considered thresholds for positive and negative correlation, respectively (Table S4). Interestingly, the *PheCAD2* gene has positive correlation with most of the lignin biosynthesis genes *PhePAL* (PH02Gene08453, PH02Gene23138, PH02Gene30508 and PH02Gene42984), *PheC4H* (PH02Gene03697), *Phe4CL* (PH02Gene34989), *PheHCT* (PH02Gene06655, PH02Gene25625), *PheCCoAOMT* (PH02Gene02562), *PheCCR* (PH02Gene06795, PH02Gene11141, PH02Gene14706, PH02Gene25511, PH02Gene28903, PH02Gene42850, PH02Gene47460, PH02Gene48085 and PH02Gene48963) and *PheCOMT* (PH02Gene05362, PH02Gene17241) (Figure 4A). These co-expression results indicate that except for the *PheC3H*, *PheCSE* and *PheF5H* genes, all other lignin biosynthesis genes involved in producing H, S and G lignin units were co-expressed with *PheCAD2*.

Similarly, *PheCAD2* also has a negative correlation with *Phe4CL* (PH02Gene24626) (Figure 4B), whereas *PheCAD1* has a positive correlation with *PheCCR* (PH02Gene51351, PH02Gene48149) and negative correlation with *Phe4CL* (PH02Gene09462) and *PheCCR* (PH02Gene04803, PH02Gene28140, PH02Gene41539). Further, *PheCAD8* has a positive correlation with *PheCAD7* (PH02Gene32740) and a negative correlation with *Phe4CL* (PH02Gene00825). At the same time, *PheCAD7* has a positive correlation with *Phe4CL* (PH02Gene09462), *PheCSE* (PH02Gene39642, PH02Gene49207), *PheCCR* (PH02Gene04803, PH02Gene28140). Whereas negative correlation with *PheCAD1*, *Phe4CL* (PH02Gene00825) and *PhePAL* (PH02Gene30509). Further, we also analysed the co-expression analysis of *PheCAD* genes to identify the transcription factors or regulators and other proteins involved in abiotic or biotic stress. Interestingly, more than 35% of the annotated co-expressed genes with *PheCAD* genes were involved in biotic or abiotic stress response (Table S5).

### 2.7. PheCAD Genes Response to Abiotic Stress Treatment

**Figure 5:**
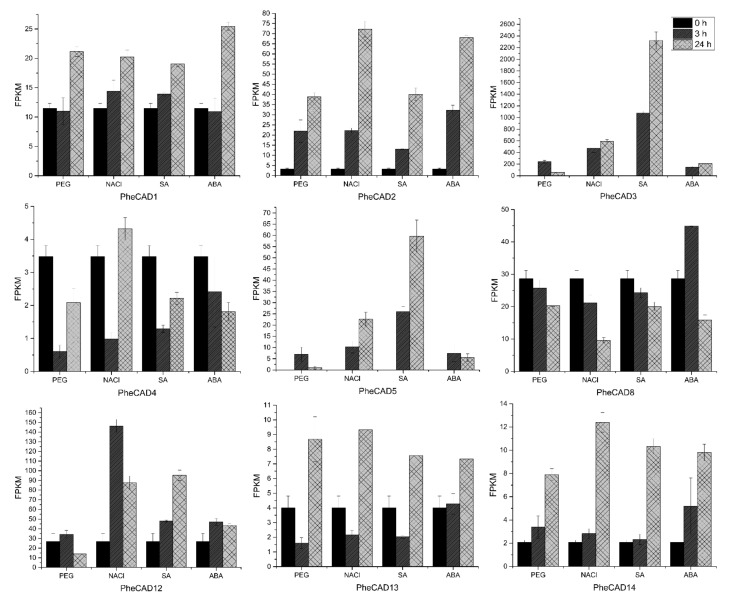
Expression analysis of *PheCAD* genes in response to PEG, NaCl, ABA and SA. The FPKM values of transcriptomic data (Moso bamboo seedlings treated with PEG (25%), NaCl (200mM), ABA (1uM), SA (1mM) for 3 and 24 h) were used to develop graphs.

Plant cells respond to abiotic stress by increasing the cell wall thickness, i.e., accumulation of hemicellulose and lignin polymer [36]. Our co-expression analysis studies also indicate that *PheCAD* genes are involved in biotic or abiotic stress responses. To identify the *PheCAD genes* involved in response to the abiotic stress, we analysed the transcriptomic data of Moso bamboo seedlings treated with SA, ABA, drought and salt (unpublished data). In this study, the genes with a two-fold change in expression compared to the control after abiotic stress treatment were considered differentially expressed. Among *PheCAD1*, *PheCAD2* and *PheCAD8* genes, the *PheCAD2* gene was significantly upregulated after all abiotic stress treatments, especially after 24 h of NaCl and ABA treatment (Figure 5).

Of other Moso bamboo *CAD* genes, *PheCAD3* and *PheCAD5* were highly upregulated after all abiotic stress treatments. After 24 h of NaCl, SA and ABA treatment, the relative expression of *PheCAD3* was upregulated to 274, 1069 and 97 times to the control, respectively. After 3 h of PEG treatment, *PheCAD3* expression was upregulated to about 111 times that of the control and decreased 29 times after 24 h PEG treatment. Further, the *PheCAD5* gene expression was found to be nil in control, but after NaCl and SA treatment, the relative expression was upregulated to about 100 and 250 after 3 h and 226 and 597 times after 24 h, respectively. Whereas after PEG and ABA treatment, the relative expression was upregulated to 70 and 74 times that of control after 3 h but decreased to 10 and 55 times after 24 h, respectively. Interestingly, both *PheCAD3* and *PheCAD5* are also very closely related in the phylogenetic tree (Figure 1). After 3 h of PEG, NaCl and SA treatment, the *PheCAD4* gene expression was downregulated 0.1–0.3 times to control. At the same time, the *PheCAD12* gene expression was upregulated 5.5 to 3.3 times of control after 3 and 24 h of NaCl treatment, respectively. In contrast, the expression was upregulated 3.6 times of control after 24 h of SA treatment. Similarly, after 3 h of PEG treatment, the relative expression of *PheCAD13* was downregulated 0.4 times that of control, where the expression was upregulated two times after 24 h of PEG and NaCl treatment. The relative expression of *PheCAD14* was upregulated 3.8–6 times of control after 24 h of all abiotic treatments (Figure 5). The expression levels of *PheCAD6*, *PheCAD7*, *PheCAD9*, *PheCAD10* and *PheCAD11* genes were too low for analysis.

### 2.8. The PheCAD Enzymes Form Only Homo-Dimers in Yeast

**Figure 6:**
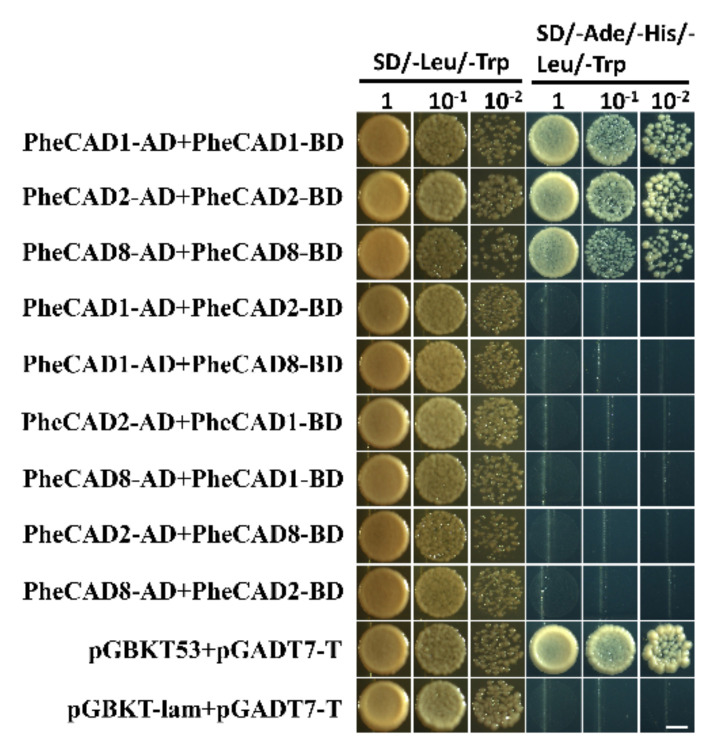
*PheCAD1*, *PheCAD2* and *PheCAD8* enzyme homo-dimers were detected with Y2H experiments. All the combinations of *PheCAD1*, *PheCAD2* and *PheCAD8* enzymes were used as both prey and bait. Yeast carrying pGBKT53, pGADT7-T used as positive control and pGBKT-lam, pGADT7-T used as a negative control. The transformed yeast cells grew on growth (SD/-Trp/-His) and selective (SD/-Ade/-His/-Trp/-His) media. Images were taken after 5 days of incubation at 30 °C. Scale bar, 3 mm.

Lignin biosynthesis pathway enzymes form homo- and hetero-dimers and also interact with each other for metabolic channelling [32]. Further, the formation of homo- and hetero-dimers increases the catalytic efficiency compared with individual enzymes [37]. Recently Yan et al., (2019) identified PtrCAD1 forms homo-dimers in *P. trichocarpa* [33]. Moreover, it was reported that Ptr4CL, PtrC4H and PtrC3H also form homo- and hetero-dimers in *P. trichocarpa* [37,38]. Therefore, based on the tissue expression analysis, we selected the highly expressed CAD enzymes *PheCAD1*, *PheCAD2* and *PheCAD8* for Y2H experiments to confirm whether Moso bamboo CAD genes can also form the homo- or hetero-dimers. The yeast cell cotransformed with pGBKT-7-*PheCAD1* and pGADT7-*PheCAD1*, pGBKT-7-*PheCAD2* and pGADT7-*PheCAD2*, pGBKT-7-*PheCAD8* and pGADT7-*PheCAD8* and positive control grew normally on SD/–Ade/–His/–Leu/–Trp medium. The yeast cells cotransformed with other combinations, and the negative control failed to grow on SD/–Ade/–His/–Leu/–Trp medium (Figure 6). These results suggest that *PheCAD1*, *PheCAD2* and *PheCAD8* enzymes form only homo-dimers and fail to form hetero-dimers in the yeast cells.

### 2.9. Formation of Both PheCAD Homo- and Hetero-Dimers in Planta

**Figure 7:**
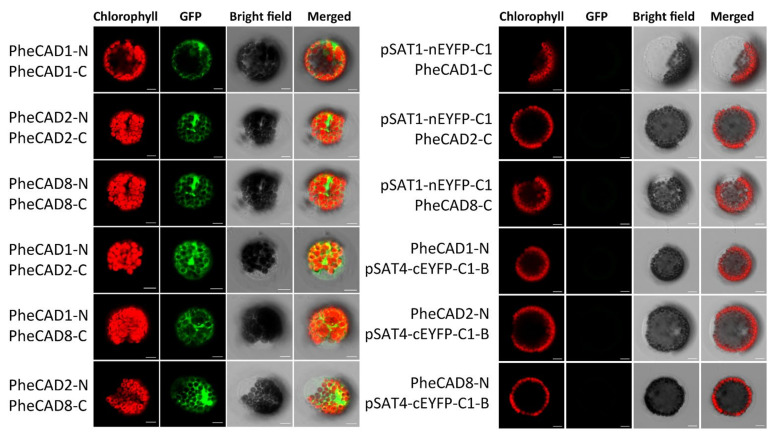
Pairwise bimolecular fluorescence complementation assay of *PheCAD1*, *PheCAD2* and *PheCAD8* enzymes in Arabidopsis protoplast cells. Empty vector and corresponding C-terminal or N-terminal PheCAD genes were used as the negative control. Scale bar, 10 μm.

Bimolecular fluorescence complementation assay (BiFC) in Arabidopsis protoplast cells was performed to confirm the Y2H results. We generated constructs containing *PheCAD1*, *PheCAD2* and *PheCAD8* genes fused with both N-terminal and C-terminal fragments of EYFP in the pSAT1-nEYFP-C1 and pSAT4-cEYFP-C1-B vectors, respectively. A strong fluorescence signal in the Arabidopsis protoplast cells was observed when pairwise cotransformation of 35S-*PheCAD1*:pSAT1-nEYFP-C1 and 35S-*PheCAD1*:pSAT4-cEYFP-C1-B; 35S-*PheCAD2*: pSAT1-nEYFP-C1 and 35S-*PheCAD2*:pSAT4-cEYFP-C1-B and 35S-*PheCAD8*:pSAT1-nEYFP-C1 and 35S-*PheCAD8*:pSAT4-cEYFP-C1-B vectors (Figure 7). These results confirmed the Y2H results of the formation of homo-dimers. At the same time, in contrast to Y2H results, a strong signal was also observed in the Arabidopsis protoplast cells when pairwise cotransformation of 35S-*PheCAD1*:pSAT1-nEYFP-C1 and 35S-*PheCAD2*:pSAT4-cEYFP-C1-B; 35S-*PheCAD1*:pSAT1-nEYFP-C1 and 35S-*PheCAD8*:pSAT4-cEYFP-C1-B; 35S-*PheCAD2*:pSAT1-nEYFP-C1 and 35S-*PheCAD8*:pSAT4-cEYFP-C1-B vectors into Arabidopsis protoplast cells (Figure 7). These results indicate that *PheCAD1*, *PheCAD2* and *PheCAD8* enzymes interact with each other and can form hetero-dimers in planta.

### 2.10. Confirmation of PheCAD Homo-Dimers and Hetero-Dimers through Co-Immunoprecipitation Experiments

**Figure 8:**
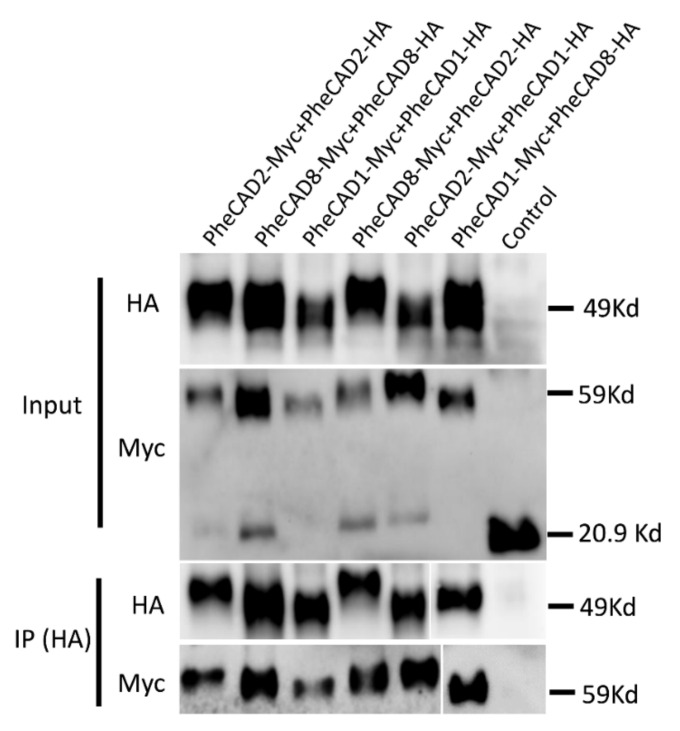
CO-IP assay of *PheCAD1*, *PheCAD2* and *PheCAD8* enzymes using proteins expressed in *N. benthamiana* leaf epidermal cells BiFC assay. The proteins were immunoprecipitated with anti-HA antibodies and detected with both anti-HA and anti-Myc antibodies.

To further confirm the PheCAD homo- and hetero-dimers through co-immunoprecipitation coupled with immunoblotting, SPYNE173 (Myc-tag) and SPYCE(M) (HA-tag) constructs were used. Different combinations of PheCAD genes (*PheCAD1*-Myc + *PheCAD1*-HA, *PheCAD2*-Myc + *PheCAD2*-HA and *PheCAD8*-Myc + *PheCAD8*-HA, *PheCAD2*-Myc + *PheCAD1*-HA, *PheCAD1*-Myc + *PheCAD8*-HA and *PheCAD8*-Myc + *PheCAD2*-HA) were injected into tobacco leaves. The total protein was isolated from the injected leaves, and immunoprecipitation experiments were conducted with anti-HA-antibodies to pull down the HA fusion proteins. If HA tag protein interacts with Myc tag protein, the HA beads should also pull down the Myc-tag protein. Both total protein and the purified protein was subjected to Western blotting using anti-HA and anti-Myc antibodies. The detection of bands with anti-Myc antibodies in pull-down assay confirmed that the *PheCAD1*, *PheCAD2*, and PheCAD3 enzymes form homo-dimers, and these proteins interact with each other and form hetero-dimers in planta (Figure 8). The uncropped Western blot figures were provided as a Supplementary Materials.

## 3. Discussion

Lignin is the major structural component of the secondary cell wall that provides mechanical strength for the upright growth of vascular plants and provides rigidity to the cell wall [39]. It interacts with cellulose and hemicellulose and helps in effective water transport by creating a hydrophobic environment in the vascular bundle. However, lignin renders the cell wall processability and is the primary barrier for biofuel and paper production [40]. Bamboos are the fastest-growing plants that are useful for food, timber and paper production. Thus, studying lignin biosynthesis in bamboo is not only crucial for understanding plant biology but also for genetic engineering strategies to improve pulp quality for paper production. CAD is the key rate-limiting enzyme in the lignin biosynthesis involved in the catalysis of coniferaldehyde, p-coumaraldehyde, and sinapaldehyde to corresponding alcohols and also for biotic and abiotic stress response [41]. The *CAD* gene family is investigated in so many plant species’ Arabidopsis [7,42]: rice [43], wheat [44], sorghum [45], *P. trichocarpa* [46], sweet potato [17], *Brachypodium distachyon* [6], tea [21], melon [41] and pear [47], etc. The present study aims to identify *CAD* genes in available Bambusoideae genome sequences and functional characterisation of Moso bamboo *CAD* genes.

In *Angiosperms*, *CAD* genes have distinct roles and are expressed in different stages and tissues during plant growth and development [17,41]. In Arabidopsis, *AtCAD4*, *AtCAD5*, *AtCAD7* and *AtCAD8* genes were involved in lignin biosynthesis. Among them, *AtCAD4* was strongly expressed in roots and flowers, whereas *AtCAD5* was in lignified roots [7,15]. Similarly, *BdCAD5* in *B. distachyon* was expressed in most tissues and highest in stem and root. At the same time, the *BdCAD3* expression was confined to stem and spikes [6]. In melon, *CmCAD1* was expressed from 25–33 days after anthesis, whereas *CmCAD2* and *CmCAD5* genes were strongly expressed during fruit development. The *CmCAD5* is also expressed in most vegetative tissues, with the highest in flower and negligible in mature leaves [41]. Similarly, this study identified that *PheCAD2* had intense expression in most tissues except leaves, root rhizome, root on shoot—2 cm and 6.7m height shoot, especially *PheCAD8* expressed in the tissues where the *PheCAD2* gene expression was absent. *PheCAD1*, *PheCAD6* and *PheCAD12* were also expressed in most tissues, indicating that these five *PheCAD* genes might be involved in lignification (Figure 3).

It has been demonstrated that some of the lignin biosynthesis enzymes are loosely or transiently associated with each other to form metabolic channelling or metabolon and also form homo- or hetero- dimers. The early steps of lignin biosynthesis enzymes such as PAL isoforms in tobacco cells associate with C4H and substrate channel for better catalytic efficiency and regulation of metabolic flux [48,49,50]. In Arabidopsis, the cytochrome P450 enzymes of lignin biosynthesis (AtC4H, AtC3H and AtF5H) were found to associate with each other to form an enzyme complex or a metabolon with the help of membrane steroid-binding proteins (AtMSBPs) [51,52]. Further, in *P. trichocarpa*, the isozymes of PtrC4H, PtrC3H form hetero-dimeric (PtrC4H1/C4H2, PtrC4H1/C3H3, and PtrC4H2/C3H3) and heterotrimeric (PtrC4H1/C4H2/C3H3) enzyme complexes and increase the catalytic efficiency of the complex by 70–6500 fold as compared with individual enzymes [37]. Moreover, Chen et al., (2013, 2014) [38,53] have demonstrated that Ptr4CL3 and Ptr4CL5 isoforms interact to form a tetramer both in vivo and in vitro and proposed a regulatory role for it in *P. trichocarpa*. Additionally, PtrCAD1 interacts with PtrCCR2, and both enzymes were found to form homo-dimers in *P. trichocarpa* [33]. This study identified that *PheCAD1*, *PheCAD2* and *PheCAD8* enzymes interact and form homo-dimers in yeast (Figure 6) and both homo- and hetero- dimers in planta (Figure 7 and Figure 8). The failure to form hetero- dimers in yeast is due to post-translation modifications and the unavailability of other components, which might be needed in the formation of hetero-dimers. These results and the above literature indicate that PheCCR might interact with the PheCAD homo- or hetero- dimers. Therefore, studying the CAD homo- and hetero- dimers and PheCCR ratio within the plants and the catalytic efficiency would be interesting.

Besides lignification, *CAD* genes are also involved in biotic and abiotic stress. Previous studies reported that *CAD1* gene expression is strongly induced by both biotic and abiotic stress in sweet potatoes and *Ginkgo biloba* [17,19]. Similarly, the oriental melon *CmCAD2* gene was significantly induced after salt and drought stress [22]. Further, in Arabidopsis, among the most expressed *CAD* genes *AtCAD-C* and *AtCAD-D*, only *AtCAD-D* was strongly induced after biotic stress. Whereas among the least active *CAD* genes, *AtCAD-B1* was induced in response to biotic stress [15]. Similarly, our investigation of moso bamboo *PheCAD* genes in response to abiotic stress identified that *PheCAD2* was significantly induced after all the abiotic stress treatments among the highly expressed *CAD* genes. These results indicate involvement in both lignification and also in defence of the abiotic stress response. Among least-expressed *PheCAD* genes (*PheCAD3*, *PheCAD4*, *PheCAD5*, *PheCAD7*, *PheCAD9*, *PheCAD10*, *PheCAD11*, *PheCAD13* and *PheCAD14*), *PheCAD3* and *PheCAD5* genes were induced tremendously after all abiotic stress treatments (Figure 5). These results indicate the involvement of *PheCAD3* and *PheCAD5* genes only in response to abotic stress. In conclusion, our results identified the major *PheCAD* genes involved in the lignin biosynthesis pathway and abiotic stress response. Further, identifying homo- and hetero-dimers of the PheCAD enzymes provides new evidence that PheCAD enzymes might act as a complex in a tissue-specific manner.

## 4. Materials and Methods

### 4.1. Identification of CAD Enzymes from Bambusoideae Genome Database

The CAD enzymes amino acid sequences of rice were downloaded from Oryzabase (https://shigen.nig.ac.jp/rice/oryzabase/, acessed on 1 November 2021), and these sequences were used to identify CAD enzymes from the Bambusoideae genome database. The Moso bamboo CAD enzymes were identified from the *P. edulis* genome database through local BLASTP [30]. Similarly, for herbaceous diploid bamboo species *R. guianensis* and *O. latifolia*, the tetraploid and hexaploid woody species *G. angustifolia* and *B. amplexicaulis* CAD enzymes were identified from available draft genomes [31]. The sequences containing the catalytic and structural Zn-binding and NADP(H) binding sites were considered CAD enzymes.

### 4.2. Phylogenetic Tree and Motif Analysis of CAD Enzymes

The phylogenetic tree was constructed in the MEGA-X software using both a maximum likelihood and a neighbour-joining method [35]. The aligned peptide sequences of *A. thaliana*, *P. trichocarpa*, *Oryza sativa*, *O. latifolia*, *R. guianensis*, *G. angustifolia, P. edulis*, and *B. amplexicaulis* were used to generate a phylogenetic tree (Table S1). To access the statistical significance of the clades in the phylogenetic tree, a bootstrap value of 1000 replicates was used. The conserved motifs were identified through the MEME server and visualised in TBtools [54].

### 4.3. Expression Analysis

We downloaded the transcriptomic data of 26 different kinds of tissues published by Zhou et al. (2018) [30] through the NCBI Short Read Archive database (SRX2408703). We analysed these data, and the FPKM values of the Moso bamboo *CAD* genes were used to develop a heatmap by TBtools [54]. For abiotic stress treatment, 30-day-old Moso bamboo seedlings with similar height were used. The seedlings were treated individually with 200 mM sodium chloride (NaCl), 25% polyethylene glycol (PEG), 1mM salicylic acid (SA) and 1uM abscisic acid (ABA) nutrient solution for 3 h and 24 h. Total RNA was isolated from young leaves, and transcriptomic data were generated in three biological replicates (NCBI ID: GSE169067). The graphs were developed using FPKM values of the *PheCAD* genes transcriptomic data.

### 4.4. Co-Expression Analysis

The FPKM values of 26 different kinds of transcriptomic tissue data developed by Zhou et al., (2018) [30] were used for the co-expression analysis. A minimum threshold of 0.05 FPKM value in more than six tissues out of 26 tissues was chosen as a cutoff to identify lignin biosynthesis genes. The Pearson correlation coefficient (PCC) was calculated, and the gene pairs with PCC values 0.7 to 1 and −0.5 to −1were considered to have positive and negative correlation, respectively. These gene pairs were visualised using Cytoscape. Further to identify total genes co-expressed with *PheCAD* genes, we submitted the *PheCAD* genes to the BambooNET (http://bioinformatics.cau.edu.cn/bamboo/index.html, accessed on 1 November 2021) and developed the co-expression network.

### 4.5. Yeast Two-Hybrid (Y2H) Assay

The coding sequences of *PheCAD* genes were amplified by using specific primers designed from 5′ UTR and 3′ UTR using leaf and stem cDNA as a template. This purified full-length PCR product was used to amplify adapter containing gene-specific primers (Table S6) and cloned into the pGBKT-7 and pGADT7 vectors (Clontech, Beijing, China). The recombinant vectors pGBKT-7 and pGADT7 containing *PheCAD1*, *PheCAD2* and *PheCAD3* genes were cotransformed into AH109 yeast strain following the manufacturer’s instructions (Matchmaker^TM^ GAL4 two-hybrid system, Clontech). The vectors pGBKT53+pGADT-Lam and pGBKT-53+pGADT7-T were used as negative and positive controls, respectively. The transformed yeast cells were cultured on SD/-Leu/-Trp with Agar medium (Coolaber, Beijing, China) at 30 °C for three days. The colonies grown in this medium were selected, dissolved into ddH_2_o and plated onto the selection plates containing SD/-Ade/-His/-Leu/-Trp medium (Coolaber, Beijing, China) as serial dilutions; the plates were incubated at 30 °C for 5 days. The pictures were taken on the 5th day under a light microscope.

### 4.6. Bimolecular Fluorescence Complementation Assay in Arabidopsis Protoplast

The three *PheCAD* genes were cloned into pSAT1- nEYFP-C1 and pSAT4-cEYFP-C1-B vectors. The recombinant YFPN and YFPC fusion vectors transiently co-expressed in mesophyll protoplasts of Arabidopsis, according to Yoo et al., (2007) [55]. The transformed cells images were captured in a confocal microscope at wavelength 488 and 594 nm argon laser.

### 4.7. Co-Immunoprecipitation and Immunoblot Analysis

To generate pull-down constructs, the three *PheCAD* genes were cloned under the 35S promoter of SPYNE173 and SPYCE(M) vectors (contain Myc- and HA- tag, respectively). The recombinant vectors and empty vectors were transformed into *Agrobacterium* strain GV3101. Overnight cultures were collected and resuspended in buffer containing 10 mM MgCl2, 10 mM MES-K (pH 5.6) and 100 μM acetosyringone to OD600 nm at 0.4. The resuspended agrobacterium solutions carrying Myc- and HA- tag vectors combined in 1:1 ration and then co-infiltrated into the abaxial side of *N. benthamiana* 4-week-old leaves. On the 3rd day after the infiltration, the total protein from tobacco leaves was extracted using Plant Protein Extraction Reagent (CWBIO, Beijing, China). An equal amount of total protein was taken as input for CO-IP experiments. Pierce Anti-HA magnetic beads (Thermo Scientific, Waltham, MA, USA) were used to immunoprecipitate HA-tagged proteins as per the manufacturer’s instructions. The proteins were separated on SDS-PAGE gels (TGX Stain-Free FastCast Acrylamide Kit, 12% [Biorad, Hercules, CA, USA]) and blotted onto PVDF membrane (0.45 μm, Merck Millipore, Burlington, VT, USA). The HA and Myc fusion proteins were detected using mouse anti-HA (Affinity Biosciences, Cincinnati, OH, USA) and rabbit anti-Myc (Hangzhou HuaAn Biotechnology Co., Ltd., Hangzhou, China) antibodies. The dilutions of antibodies were used as per the instructions of the manufacture. The bands were visualised in ChemiDoc^TM^ MP Imaging System (Biorad, Hercules, CA, USA) by chemiluminescence assay (The ECL^TM^ Prime Western Blotting Detection Reagent kit, Amersham^TM^, Amersham, UK).

## Data Availability

SRX2408703, GSE169067. All the data pertaining to this study can be accessed from the lead authors on reasonable request.

## Supplementary Materials

The following are available online at https://www.mdpi.com/article/10.3390/ijms222312917/s1.
