# Supplementary material for: Homo- and Hetero-Dimers of CAD Enzymes Regulate Lignification and Abiotic Stress Response in Moso Bamboo
Source: Int J Mol Sci. 2021 Nov 29;22(23):12917. doi: 10.3390/ijms222312917 (PMC8657895; doi:10.3390/ijms222312917)
Supplement: Supplementary file 1 [file ijms-22-12917-s001.zip › Supplementary files/File S1.pdf]

File S1: Alignment file of all CAD enzymes used in this study

```
                                                   GHEXXGXXXXV          GDXXXXXXXXXCXXCXXXX
Rgu021554                       SDMFAKEGVMPATPFPIVPGHEVVGLVDAVGEGVLGSWSAGDRVGVGWFGGACYHCDRCR 99
Gan031993                       ADVIWTQNKHNDSMYPLVPGHEIVGVVRDVGSDVK-CFKVGDHVGVGTYVNSCGDCENCN 122
OsCAD4_LOC_Os11g40690.1         ADVIWTRNMFNDSIYPLVPGHEIAGVVTEVGADVK-GFKVGDH-----------DCENCN 96
Bam015552.1                     GDVIWTRNKHNDSMYPLVPGHEIAGVVTEVGSEVK-GFKVGDHVGVGTYVNSCQDCENCN 114
OsCAD1.1_LOC_Os10g11810.1       ADVAWTRNILNNSMYPLVPGHEIAGVVTEVGADVK-SFKVGDHVGVGTYVNSCRDCENCN 107
OsCAD1.3_LOC_Os10g11810.3       ---------MKLCLNFNMSRHEIAGVVTEVGADVK-SFKVGDHVGVGTYVNSCRDCENCN 50
Ola036924.1                     ADVAWTRNMLNNSMYPLVPGHEIAGVVTEVGSDVK-GFKVGDHVGVGTYVNSCRDCEYCD 107
Rgu012272                       ADVAWTRNMLNNSMYPLVPGHEIAGVVTEVGSNVK-GFKVGDHVGVGTYVNSCRDCECCN 107
Bam025746.1                     ADVAWTRNMHTDSMYPLVPGHEIAGVVTEVGSAVE-RFKVGDHVGVGTYVNSCRDCENCN 197
PH02Gene48685.t1                ADVAWTHNMLNNSMYPLVPGHEIAGVVTEVGSDVK-GFKVGDHVGVGTYVNSCRDCEICN 107
PH02Gene44682.t1                ADVVWTRNMHNDSMYPLVPGHEIAGVVTEVGSDVK-GFKVGDHVGVGTYVNSCRDCESCN 107
PH02Gene46268.t1                ADVVWTRNMHNDSMYPLVPGHEIAGVVTEVGSDVK-GFKVGDHVGVGTYVNSCRDCESCN 107
Ola035994.1                     TDIHQAKNDLGASKYPMVPGHEVVGEVVEVGPEVT-KFRAGEVVGVGVIVGCCRQCRPCK 105
Rgu003339                       TDIHQAKNDLGASKYPMVPGHEVVGEVMEIGPEVT-KCRAGDVVGVGVIVGCCRECRPCK 77
OsCAD2-1_Os02t0187800-01        TDIHQAKNHLGASKYPMVPGHEVVGEVVEVGPEVT-KYSAGDVVGVGVIVGCCRECHPCK 107
OsCAD2-2_Os02t0187800-02        TDIHQAKNHLGASKYPMVPGHEVVGEVVEVGPEVT-KYSAGDVVGVGVIVGCCRECHPCK 66
Gan017720                       TDIHQAKNELGGSKYPMVPGHEVVGEVVEVGPEVS-RYRAGDIVGVGVIVGCCRECRPCK 98
Bam010499.1                     TDIHQAKNELGASKYPMVPGHEVVGEVEEVGPEVT-KYRAGDVVGVGVIVGCCRECRPCK 107
PH02Gene39617.t1                TDIHQAKNELGASKYPMVPGHEVVGEVVEVGPDVT-RYRAGDVVGVGVIVGCCRECHPCK 107
PH02Gene04778.t1                TDIHQAKNELGASKYPMVPGHEVVGEVVEVGPEVT-KYRAGDVVGVGVIVGCCRECRPCK 107
Bam007854.1                     TDIHQAKNELGASKYPMVPGHEVVGEVVEVGPEVT-KYRAGDVVGVGVIVGCCRECRPCK 107
OsCAD6_LOC_Os04g15920.1         TDLHFIHNDWGITMYPVVPGHEITGVVTKVGTNVA-GFKVGDRVGVGCIAASCLDCEHCR 108
Ola016985.1                     TDLHFIHNDWGITMYPVVPGHEITGVVTKVGTNVS-GLKAGDRVGVGCIAASCLDCEHCR 92
Bam039144.1                     SDLHFIHNDWGITMYPVVPGHEITGIVTKVGTNVS-GFKVGDRVGVGCIAASCLDCEHCR 92
Bam034885.1                     SDLHFIHNDWGITMYPVVPGHEITGIVTKVGTNVS-GFKVGDRVGVGCIAASCLDCEHCR 108
Bam039795.1                     TDLHFIHNDWGITMYPVVPGHEITGIVTKVGTNVS-GFKVGDRVGVGCIAASCLDCEHCR 108
PH02Gene48202.t1                SDLHFIHNDWGITMYPVVPGHEITGIVTKVGTNVS-GFMVGDRVGVGCIAASCLDCEHCR 108
PH02Gene49901.t1                TDLHFIHNDWGITMYPVVPGHEITGIVTKVGTNVS-GFMVGDRVGVGCIAASCLDCEHCR 108
Gan014390                       TDLHFIHNDWGITMYPVVPGHEITGMVTKVGTNVS-GFMVGDRVGVGCIAASCLDCEHCR 108
Rgu008819                       SDLSTIKNEWGNAKYPVVPGHEIVGVVTDVGPSVT-RFKVGDNVGVGYMASACRDCANCQ 113
OsCAD9_LOC_Os03g12270.1         SDLSTIKNEWGNAKYPVVPGHEIVGVVAEVGSSVA-RFAAGDTVGVGYIASTCRACANCR 105
Gan006403                       ----------------------MVGVVAEVGSSVT-RFNVGDKVGVGYIASTCRACPSCQ 37
Ola001888.1                     SDLSTIKNEWGNAKYPVVPGHEIVGVVTDVGASVT-RFKAGDNVGVGYIASACRACTNCL 104
PH02Gene26598.t1                TDLSTIKNEWGNAKYPVVPGHEIVGVVTEVGSSVT-RFKAGDKVGVGYIASTCRACPNCQ 104
Bam045387.3                     SDLSTIKNEWGNAQYPVVPGHEIVGVVTEVGSSVT-RFKAGDKVGVGYIASTCRACPNCQ 712
OsCAD8B_LOC_Os09g23540.1        TDLHVVKNDWGNAMYPVVPGHEIVGVVTGVGAGVT-KFKAGDTVGVGFFVGSCRTCDSCG 180
OsCAD8C1_LOC_Os09g23550.1       TDLHIVKNDWGNAMYPVVPGHEIVGVVTGVGAGVT-KFKAGDTVGVGYFVASCRGCECCG 180
OsCAD8C2_LOC_Os09g23550.2       -------------MYPVVPGHEIVGVVTGVGAGVT-KFKAGDTVGVGYFVASCRGCECCG 46
Ola032369.1                     TDLHVIKNDWGNSLYPVVPGHEILGIVADVGSGVK-KFKAGDTVGVGYFVGSCRSCESCG 104
Rgu014373                       TDLHVIKNDWGNALYPVVPGHEIVGVVAGVGSGVK-KFKAGDTVGVGYFVGSCRSCESCG 105
Gan002573                       TDLHIIKNDWGNALYPVVPGHEILGVVVDVGSGVK-KFKAGDTVGVGYFVGSCRSCECCR 79
Gan004029                       TDLHIIKNDWGNALYPIVPGHEIVGVVADVGSGVK-KFKAGDTVGVGYFVGSCRSCECCG 77
PH02Gene13789.t1                TDLHIIKNDWGNALYPVVPGHEILGVVGDVGSGVK-KFKAGDTVGVGYFVGSCRSCECCG 104
Bam027739.1                     TDLHIIRNDWGNALYPVVPGHEILGVVAGVGSGVK-KFKAGDTVGVGYFVGSCRSCECCG 159
OsCAD8A_LOC_Os09g23530.1        TDLHTIKNEWGNAMYPVVPGHEIVGVVAGVGAGVT-RFKAGDTVGVGYFVDSCRACDSCG 105
OsCAD8D_LOC_Os09g23560.1        TDLHIIKNEWGNAMYPVVPGHEIVGVVTGVGAGVT-KFKAGDTVGVGYFVDSCRACDSCG 105
Bam027740.1                     TDLHIIKNEWGNAMYPVVPGHEVVGIVTDVGRGVK-NFKTGETVGVGYFVDSCRTCESCS 105
Bam040591.1                     TDLHIIKNEWGNALYPVVPGHEVVGIVTDVGRGVK-NFKAGETVGVGYFVDSCRTCESCS 104
PH02Gene13790.t1                SDLHTIKNEWGNAMYPVVPGHEVVGIVTDVGRSVK-NFKAGETVGVGYFVDSCRTCESCS 219
PH02Gene13791.t1                TDLHTIKNEWSNAMYPVVPGHEVVGIVTDVGRGVK-NFKAGETVGVGYFVDSCRTCESCS 105
OsCAD3_LOC_Os10g29470.1         SDLHCIKNEWKHSIYPLVPGHEIAGVVTEVGKNVT-RFKAGDRVGVGCMVNSCRSCESCN 113
OsCAD5_LOC_Os08g16910.1         SDLHSIKNEWKNATYPLVPGHEIAGVVTEAGKNVT-KFKGGDKVGVGCMVNSCHSCDSCN 103
Rgu015472                       SDLHSIKNEWKNTIYPIVPGHEIAGVVTEVGKNVT-KFTAGEKVGVGCMVNSCHSCESCS 126
Ola032840.1                     SDLHSIKNDWKNTIYPIVPGHEIAGVVTEVGKNVT-RLNVGDKVGVGCIVNSCQSCESCN 114
PH02Gene38471.t1                SDLHSIKNEWKNAIYPIVPGHEIAGVVTEVGKNVT-KFKAGDKVGVGCMVNSCQSCESCN 113
Bam005700.1                     SDLHSIKNDWKNAIYPIVPGHEIAGVVTEVGKNVT-KFKAGDKVGVGCMVNSCQSCESCN 114
Bam017920.1                     SDLHSIKNDWKNAIYPIVPGHEIAGVVTEVGKNVT-KFKAGDKVGVGCMVNSCQSCESCN 114
PH02Gene38470.t1                SDLHSIQNEWKNAIYPIVPGHEIAGVVTEVGNNVT-KFKAGDKVGVGCMVNSCQSCESCN 111
Bam032152.1                     SDLHIIKNEWKNTVYPIVPGHEIAGVVTEVGKNVT-NFRAGDKVGVGCMVNSCQSCGSCN 114
Gan021750                       SDLHSIKNEWKNAIYPIVPGHEIAGVVTEVGKNVT-KFKAGDKVGVGCMVNSCQSCDNCN 66
Gan024020                       SDLHSIKNEWKNAIYPIVPGHEIAGVVTEVGKNVT-KFKAGDKVGVGCMVNSCQSCDNCN 77
OsCAD7_Os04t0612700-01          SDLHTIKNEWRNAVYPVVAGHEITGVVTEVGKNVA-RFKAGDEVGVGCMVNTCGGCESCR 120
Ola022375.1                     SDLHTIKNEWKNAIYPVVPGHEIVGVIAEVGEKVE-RFKAGDKVGVGCMVNTCQSCESCK 116
Bam022082.1                     SDLHTIKNEWRSAIYPVVAGHEIVGLINEVGKNVQ-EFKVGDKVGVGCMVNTCQSCESCK 115
PH02Gene32740.t1                SDLHTIKNEWRNATYPVVAGHEIVGLITEVGKNVQ-MFKAGDKVGVGCMVNTCQSCESCK 113
Bam044253.1                     SDLHTIKNEWRNAIYPVVAGHEIVGLITEVGKNVQ-KFKAGDKVGVGCMVNTCQSCESCK 111
Rgu006342                       TDLHHLRIT-ADSVFPLVPGHEFVGEVAAVGEEVT-AHAVGDPVAVGNIVDSCGECRRCR 78
Rgu008067                       SDIHQVRGEWGGETFPMVPGHEIIGTVAEVGSDVT-EHAVGDTVGVGCFVDSCLECEACK 180
Rgu021228                       SDIHQARGEWGGESFPMVPGHEIVGEVVEVGSAVS-GHAVGDLVGVGCFVDSCLECEACQ 112
Rgu020259                       SDIHTVKGDWGPQPFPVVPGHEIVGRVAEVGDEVT-THRVGDRVGVGCLVNSCRECVNCR 102
Rgu021242                       SDIHQARNEWGGSEFPMVPGHEIAGTVSAVGAEVS-QYAVGDRVGVGCFVDSCRECDNCR 109
                                                      . * :   *  *      *:             *  *
```

```
                               XXXE
Rgu021554                      RGDFITCENGRI-----------PGVTFDGGYAEALVVPADALARVPD--------ELGA 140
Gan031993                      SFLENYCSN-TVFTFNRID---TDGSVTMGGYSNHIVVRQRYCFKIPD--------GYPL 170
OsCAD4_LOC_Os11g40690.1        SSLENHCSK-CVVTYNSVD---SDGTVTKGGYSSHILVHQRYCFKIPA--------DYPL 144
Bam015552.1                    SFLENHCSK-FVFTYNSID---TDGTVTKGGYSSHIVVHERYCHKIPD--------GYPL 162
OsCAD1.1_LOC_Os10g11810.1      SSLENYCSQ-HVFTFNGVD---TDGTVTKGGYSTHIVVHERYCFKIPD--------GYPL 155
OsCAD1.3_LOC_Os10g11810.3      SSLENYCSQ-HVFTFNGVD---TDGTVTKGGYSTHIVVHERYCFKIPD--------GYPL 98
Ola036924.1                    SFLENHCSK-FVFTFNGVD---TDGAVTKGGYSTHIVVHERYCFKIPD--------GYPL 155
Rgu012272                      SFLENHCSN-FVFTFNGID---ADGTVTKGGYSTHIVVHERYCFKIPD--------GYPL 155
Bam025746.1                    SFLEIHCSN-FVFTFNGVD---TDGTVTKGGYSTHIVVHERYCFKIPD--------GYPL 245
PH02Gene48685.t1               SFLENHCSK-HVFTFNGVD---TDGTVTKGGYSTHIVVHERYCFKIPD--------GYPL 155
PH02Gene44682.t1               SFLEVHCSK-FVSTFNGVD---TDGTVTKGGYSTHIVVHKRYCFKIPD--------GYPL 155
PH02Gene46268.t1               SFLENHCSK-FVFTFNGVD---TDGTVTKGGYSTHIVVHERYCFKIPD--------GYPL 155
Ola035994.1                    ANVEQYCNK-RIWSYNDVY---TDGRPTQGGFSSAMVVDQKFVVKVPD--------GLAP 153
Rgu003339                      ANVEQYCNK-RIWSYNDVY---TDGRPTQGGFSSAMVVDQKFVVKVPA--------GLAP 125
OsCAD2-1_Os02t0187800-01       ANVEQYCNK-RIWSYNDVY---TDGRPTQGGFASAMVVDQKFVVKIPA--------GLAP 155
OsCAD2-2_Os02t0187800-02       ANVEQYCNK-RIWSYNDVY---TDGRPTQGGFASAMVVDQKFVVKIPA--------GLAP 114
Gan017720                      ANVEQYCNK-KIWSYNDVY---TDGRPTQGGFSSAMVVDQKFVVKVPA--------GLAP 146
Bam010499.1                    ANVEQYCNK-RIWSYNDVY---TDGRPTQGGFASTMVVDQKFVVKVPA--------GLAP 155
PH02Gene39617.t1               ANVEQYCNK-RIWSYNDVY---TDGRPTQGGFSSAMIVDQKFVVKVPV--------GLAP 155
PH02Gene04778.t1               ANVEQYCNK-KIWSYNDVY---TDGRPTQGGFSSAMVVDQKFVVKIPV--------GLAP 155
Bam007854.1                    ANVEQYCNK-KIWSYNDVY---TDGRPTQGGFSSAMIVDQKFVVKVPA--------GLAP 155
OsCAD6_LOC_Os04g15920.1        RSEENYCDK-VALTYNGIF---WDGSITYGGYSGMLVAHKRFVVRIPD--------TLPL 156
Ola016985.1                    RSEENYCDK-VTLTYNGIF---WDGSVTYGGYSSMLVAHKRFVVRIPD--------NLPL 140
Bam039144.1                    RSEENYCDK-VALTYNGIF---WDGSVTYGGYSNMLVAHKRFVVRIPD--------NLPL 140
Bam034885.1                    RSEENYCDK-VALTYNGIS---WDGSVTYGGYSNMLVAHKRFVVRIPD--------NLPL 156
Bam039795.1                    RSEENYCDK-VTLTYNGIF---WDGSVTYGGYSNMLVAHKRFVVRIPD--------NLPL 156
PH02Gene48202.t1               SSEENYCDK-VTLTYNGIF---WDGSVTYGGYSNMLVSHKRFVVRIPD--------NLPL 156
PH02Gene49901.t1               SSEENYCDK-VTLTYNGIF---WDGSVTYGGYSNMLVAHKRFVVRIPA--------NLPL 156
Gan014390                      RSEENYCDK-VTLTYNGIF---WDGSVTYGGYSNMLVAHKRFVVRIPD--------NLPL 156
Rgu008819                      EGFENYCAG-LVPSFNSAL---PDAAAVHGGFSELAVVHEHFVLRIPG--------ASPL 161
OsCAD9_LOC_Os03g12270.1        DGFENYCAG-LVPSFNAAL---PDGATVHGGFSELAVVNQRYVVRIPG--GGGGASPAPL 159
Gan006403                      EGFENYCAG-LVPSFNSAL---PDGTKVHGGFSELAVVHEHFVLRIPD--------ASPL 85
Ola001888.1                    EGFENYCAG-LVPSFNSAL---PDGATVHGGFSELAVVHEHFVLRIPD--------ASPL 152
PH02Gene26598.t1               EGFENYCAG-LVPSFNSAL---PDGATVHGGFSELAVVHEHFVLRIPD--------ASPL 152
Bam045387.3                    EGFENYCAG-LVPSFNSAL---PDGAKVHGGFSELAVVHEHFVLRIPD--------ASPL 760
OsCAD8B_LOC_Os09g23540.1       KGYENYCPT-MVITSNGKD---YGGAATQGGFSDAIVVNEHYVLRVPA--------GLPL 228
OsCAD8C1_LOC_Os09g23550.1      NGYENYCAK-MVTTCNGVDHDHGGGAATQGGFSDAIVVNEHYVLRVPA--------GLPL 231
OsCAD8C2_LOC_Os09g23550.2      NGYENYCAK-MVTTCNGVDHDHGGGAATQGGFSDAIVVNEHYVLRVPA--------GLPL 97
Ola032369.1                    KGYENYCSG-MVLTSNGVDQEHG-GEVTKGGFSDVMVVNEDYVVRVPD--------GLPL 154
Rgu014373                      KGYENYCSG-MVMTSNGVDKEHG-GEVTKGGFSDVMVVNEDYVVRVPD--------GLAL 155
Gan002573                      KGYEQYCSK-VVFTSNGVDHDHG-GAVTQGGFSDVMVVNQDYVVRVPD--------GLPL 129
Gan004029                      KGYENYCSE-MVLTSNGVDREHG-GAVTQGGFSDVMVVNEDYVVRVPD--------GLPL 127
PH02Gene13789.t1               KGYEQYCSR-MVLTSNGVDHDHG-GAVTQGGFSDVMVVNQDYVVRVPD--------GLPL 154
Bam027739.1                    KGYEQYCSR-MVLTSNGVDHDHG-GAVTQGGFSDVMVVNQDYVVRVPD--------GLPL 209
OsCAD8A_LOC_Os09g23530.1       KGDENYCPT-MVITSNGTD---YGGATTQGGFSDVMVVRQDYVLRVPA--------SLPP 153
OsCAD8D_LOC_Os09g23560.1       KGYENYCPT-MVITSNGTD---YGGATTQGGFSDVMVVRQDYVVRVPA--------SLPP 153
Bam027740.1                    KGYENYCPT-LVPTSNGVD---YDGATTLGGFSDVLVVNQDYLVRVPE--------SLPL 153
Bam040591.1                    KGHENYCPT-LVLTSNGVD---YDGATTQGGFSDIVVVNQDYVVRVPE--------SLPL 152
PH02Gene13790.t1               KGYGNYCPT-LVPTSNGVD---YDGATTLGGFSDVVVVNQDYVVRVPE--------SLPL 267
PH02Gene13791.t1               KGYENYCPT-LVLTSNGVD---YDGATTLGGFSDVVVVNQDYVVRVPE--------SLPL 153
OsCAD3_LOC_Os10g29470.1        NGFENHCPE-GVFTYNSVD---KDGTVTYGGYSSMVVVHERFVVMFPE--------AMPL 161
OsCAD5_LOC_Os08g16910.1        QGLENHCPG-VIFTYNSVD---KDGTVTYGGYSSMVVVHERFVVRFPE--------AMPL 151
Rgu015472                      QGFENHCPG-IILTYNSVD---LDGTVTYGGYSSMVVVHERFVVRFPD--------AMPL 174
Ola032840.1                    QGFENLCPG-IILTYNSVD---RDGTVTYGGYSSMVVVHERFVVRFPE--------AMPL 162
PH02Gene38471.t1               EGFENHCPG-IIFTYNSVD---RDGTVTYGGYSTMVVVHERFVVRFPE--------AMPL 161
Bam005700.1                    EGFENHCPG-MIFTYNSVD---RDGTVTYGGYSTMVVVHERFVVRFPE--------AMPL 162
Bam017920.1                    EGFENHCPG-MIFTYNSVD---RDGTVTYGGYSTMVVVHERFVVRFPE--------AMPL 162
PH02Gene38470.t1               EGFENHCPG-MIFTYNSVD---RDGTVTYGGYSTMVVVHERFVVRFPE--------AMPL 159
Bam032152.1                    EGFENHCPG-SIFTYNSVD---RDGTVTYGGYSTMVVVHERFVVRFPE--------AMPL 162
Gan021750                      DGFENHCPG-IIFTYNSVD---RDGTVTYGGYSTMVVVHERFVVRFPE--------AMPL 114
Gan024020                      EGFENHCPG-IIFTYNSVD---RDGTVTYGGYSTMVVVHERFVVRFPE--------AMPL 125
OsCAD7_Os04t0612700-01         DGCENYCSGGVVFTYNSVD---RDGTRTYGGYSDAVVVSQRFVVRFPSSAGGGAGAALPL 177
Ola022375.1                    EGAENYCSR-IVFTYNSRD---RDGTVTYGGYSDLVVVSERFVVRFPD--------GLPL 164
Bam022082.1                    EGSENYCSK-IVFTYNSLD---RDGTTTYGGYSDLVVVSERFVIRFPD--------SLPL 163
PH02Gene32740.t1               EGSENYCSK-IVFTYNSLD---RDGTITYGGYSDLVVVSERFVVRFPD--------GLPL 161
Bam044253.1                    EGSENYCSK-IVFTYNSLD---KDGTITYGGYSDLVIVSESFVVRFPD--------GLPL 159
Rgu006342                      EGQENWCPAYVLTYAGRDR---HDGSRTLGGYSGEYVVRERFVHARPT--------DLDP 127
Rgu008067                      DGEEQYCTKGVVQTYNDEA---YDGEKTFGGYSAQVVVRDHFVVRVPE--------GMDL 229
Rgu021228                      DGEEQFCSKGVVQTYNDQG---YDDEPTHGGYSQQVVVRDHFVVRIPD--------GVDL 161
Rgu020259                      NGDEQYCLDGSVPTYAGTD---RDGTTTQGGYSTHVVVDADFVLRVPA--------GIDP 151
Rgu021242                      AGEEQFCTKGEVQTYNGRE---YTGEQTFGGYSTQVVVDENYVLRIPE--------GVEL 158
                                     *                      **::   :         *
```

```
                                                                  GXXXGXXGLGGXGHXXVKXXKXXGXXVTVXSX
Rgu021554                      EEAAPLLCAGVTTFHALRSSPLR----PGDLVAVQGIGGLGHLGIQFAARMGCEVVAISR 196
Gan031993                      AEAAPLLCAGITVYTPMMWHNMN---QPGKSLGVIGLGGLGHMAVKFGKAFGLNVTVFST 227
OsCAD4_LOC_Os11g40690.1        SKAAPLLCAGITVYTPMIRHNMN---QPGKSLGVIGLGGLGHMAVKFGKAFGLKVTVFST 201
Bam015552.1                    AEAAPLLCAGITVYTPMMRHNMN---QHGKSLGVIGLGGLGHMAVKFGKAFGLKVTVFST 219
OsCAD1.1_LOC_Os10g11810.1      EKAAPLLCAGITVYSPMMRHNMN---QPGKSLGVIGLGGLGHMAVKFGKAFGLKVTVIST 212
OsCAD1.3_LOC_Os10g11810.3      EKAAPLLCAGITVYSPMMRHNMN---QPGKSLGVIGLGGLGHMAVKFGKAFGLKVTVIST 155
Ola036924.1                    AKAAPLLCAGITVYTPMMRHQMN---QPGKSLGVIGLGGLGHMAVKFGKAFGLKVTVFST 212
Rgu012272                      AKAAPLLCAGITVYTPMMRHKMN---QPGKSLGVIGLGGLGHMAVKFGKAFGLKVTVFST 212
Bam025746.1                    AKAAPLLCAGITVYTPMMRHNMN---QPGKSLGVIGLGGLGHMAVKFGKAFGLKVTVFST 302
PH02Gene48685.t1               AKAAPLLCAGITVYTPMMRHNMN---QPGKSLGIIGLGGLGHMAVKFGKAFGLKVTVFST 212
PH02Gene44682.t1               AKAAPLLCAGITVYTPMMRHNMN---QPGKSLGIIGLGGLGHMAVKFGKAFGLKVTVFST 212
PH02Gene46268.t1               AKAAPLLCAGITVYTPMMRHNMN---QPGKSLGIIGLGGLGHMAVKFGKAFGLKVTVFST 212
Ola035994.1                    EQAAPLLCAGVTVYSPLKHFGLM---TPGLRGGILGLGGVGHMGVKVAKSMGHHVTVISS 210
Rgu003339                      EQAAPLLCAGVTVYSPLKHFGLM---AAGLRGGILGLGGVGHMGVKVAKSLGHHVTVISS 182
OsCAD2-1_Os02t0187800-01       EQAAPLLCAGLTVYSPLKHFGLM---SPGLRGGVLGLGGVGHMGVKVAKSMGHHVTVISS 212
OsCAD2-2_Os02t0187800-02       EQAAPLLCAGLTVYSPLKHFGLM---SPGLRGGVLGLGGVGHMGVKVAKSMGHHVTVISS 171
Gan017720                      EQAAPLLCAGVTVYSPLKHFGLM---APGLRGGILGLGGVGHMGVKVAKSMGHHVTVISS 203
Bam010499.1                    EQAAPLLCAGVTVYSPLKHFGLM---SPGLRGGILGLGGVGHMGVKVAKSMGHHVTVISS 212
PH02Gene39617.t1               EQAAPLLCAGVTVYSPLKHFGLM---APGLRGGILGLGGVGHMGVKVAKSMGHHVTAISS 212
PH02Gene04778.t1               EQAAPLLCAGVTVYSPLKHFGLM---APGLRGGILGLGGVGHMGVKVAKSMGHHVTVISS 212
Bam007854.1                    EQAAPLLCAGVTVYSPLKHFGLM---APGLRGGILGLGGVGHMGVKVAKSMGHHVTVISS 212
OsCAD6_LOC_Os04g15920.1        DAAAPLLCAGITVYSPMKQHGMLQADAAGRRLGVVGLGGLGHVAVKFGKAFGLHVTVIST 216
Ola016985.1                    DAAAPLLCAGITVYSPMKQHGMLQ---AGRTLGVVGLGGLGHVAVKFGKAFGLRVTVIST 197
Bam039144.1                    DAAAPLLCAGITVYSPMKQHGMLQ---AGRRLGVVGLGGLGHVAVKFGKAFGLGVTVIST 197
Bam034885.1                    DAAAPLLCAGITVYSPMKQHGMLQ---AGRRLGVVGLGGLGHVAVKFGKAFGLRVTVIST 213
Bam039795.1                    DAAAPLLCAGITVYSPMKQHGMLQ---AGRRLGVVGLGGLGHVAVKFGKAFGLRVTVIST 213
PH02Gene48202.t1               DVAAPLLCAGITVYSPMKQHGMLQ---AGRRLGVVGLGGLGHVAVKFGKAFGLRVTVIST 213
PH02Gene49901.t1               DAAAPLLCAGITVYSPMKQHGMLQ---AGRRLGVVGLGGLGHVAVKFGKAFGLRVTVIST 213
Gan014390                      DAAAPLLCAGITVYSPMKQHGMLQ---AGRRLGVVGLGGLGHVAVKFGKAFGLRVTVIST 213
Rgu008819                      AAAAPLLCAGVTVYCPMRRLGLD---RPGLHLGVAGLGGLGHLAVKFGKAFGMKVTVVST 218
OsCAD9_LOC_Os03g12270.1        DRLAPLLCAGVTVYCPMRRLGLD---RPGVHLGVAGLGGLGHLAVKFGKAFGVKVTVIST 216
Gan006403                      ERVAPLLCAGVTVYCPMRRLGLD---RPGLHLGVAGLGGLGHLAVKFGKTFGMKVTVIST 142
Ola001888.1                    DRVAPLLCAGVTVYCPMRRLGLD---RPGLHLGVAGLGGLGHLAVKFGKAFGMKVTVVST 209
PH02Gene26598.t1               DRVAPLLCAGVTVYCPMKRLGLD---RPGLHLGVAGLGGLGHLAVKFAKAFGMKVTVIST 209
Bam045387.3                    DRVAPLLCAGVTVYCPMKRLGLD---RAGLHLGVAGLGGLGHLAVKFAKAFGMKVTVIST 817
OsCAD8B_LOC_Os09g23540.1       DGAAPLLCAGVTVYSPMVIHGLN---APGKHVGVVGLGGLGHVAVKFAKAFGMRVTVIST 285
OsCAD8C1_LOC_Os09g23550.1      DSAAPLLCAGVTVYSPMVIHGLN---APGKHVGVVGLGGLGHVAVKFAKAFGMRVTVIST 288
OsCAD8C2_LOC_Os09g23550.2      DSAAPLLCAGVTVYSPMVIHGLN---APGKHVGVVGLGGLGHVAVKFAKAFGMRVTVIST 154
Ola032369.1                    DKAAPLLCAGVTVYSPMMQHGLN---EPGKHLGVVGLGGLGHVAVKFGKAFGMKVTVIST 211
Rgu014373                      DKAAPLLCAGVTMYSPMVLHGLN---APGKHLGVVGLGGLGHVAVKFGKAFGMKVTVIST 212
Gan002573                      DKAAPLLCAGVTVYSPMMHYGLN---EPGKHLGVVGLGGLGHVAIKFGKAFGMKVTVIST 186
Gan004029                      DKAAPLLCAGVTMYSPMIHYGLN---EPGKHLGVVGLGGLGHIAVKFGKAFGMKVTVIST 184
PH02Gene13789.t1               DKAAPLLCAGVTVYSPMMHYGLN---EPGKHLGVVGLGGLGHVAVKFGKAFGMKVTVIST 211
Bam027739.1                    ERAAPLLCAGVTVYSPMMRYGLN---EPGKHLGVVGLGGLGHVAVKFGKAFGMRVTVIST 266
OsCAD8A_LOC_Os09g23530.1       DGAAPLLCAGVTVYSPMVEYGLN---APGKHLGVVGLGGLGHLGVKFGKAFGMKVTVISS 210
OsCAD8D_LOC_Os09g23560.1       DGAAPLLCAGVTVYSPMVEYGLN---GPGKHLGVVGLGGLGHLGVKFGKAFGMKVTVISS 210
Bam027740.1                    DGAAPLLCAGVTVYSPMIQFGLN---APGKHLGIVGLGGLGHMAVKFGKAFGMTVTVISS 210
Bam040591.1                    DGAAPLLCAGVTVYSPMMQYGLN---APGKHLGVVGLGGLGHVAVKFGKAFGMTVTVISS 209
PH02Gene13790.t1               DGAAPLLCAGVTVYSPMMQFGLN---APGKHLGVVGLGGLGHMAVKFGKAFGMTVTVISS 324
PH02Gene13791.t1               DGAAPLLCAGVTVYSPMMQFGLN---APGKHLGVVGLGGLGHTAVKFGKAFGMTVTVISS 210
OsCAD3_LOC_Os10g29470.1        DVGAPLLCAGITVYTPMKYHGLN---APGKHVGVLGLGGLGHVAVKFARAFGLKVTVISS 218
OsCAD5_LOC_Os08g16910.1        DKGAPLLCAGITVYSPMKYHGLN---VPSKHVGVLGLGGLGHVAVKFAKAFGMTVTVISS 208
Rgu015472                      DKGAPLLCAGITVYSPMKYHGLN---TPGKHVGVLGLGGLGHVAVKFAKAFGTKVTVISS 231
Ola032840.1                    DKGAPLLCAGITVYSPMKHHRLN---SPGKHVGVLGLGGLGHVAVKFAKAFGMKVTVISS 219
PH02Gene38471.t1               DKGAPLLCAGITVYSPMKYHGLN---APGKHVGVLGLGGLGHVAVKFAKAFGMKVTVISS 218
Bam005700.1                    DKGAPLLCAGITVYSPMKYHGLN---APGKHVGVLGLGGLGHVAVKFAKAFGMKVTVISS 219
Bam017920.1                    DKGAPLLCAGITVYSPMKYHGLN---APGKHVGVLGLGGLGHVAVKFAKAFGMKVTVISS 219
PH02Gene38470.t1               DKGAPLLCAGITVYSPMKYHGLN---EPGKHVGMLGLGGLGHVAVKFAKAFGMKVTVISS 216
Bam032152.1                    DKGAPLLCAGITVYSPMKYHGLN---VPGKHIGVLGLGGLGHVAVKFAKAFGMKVTVISS 219
Gan021750                      DKGAPLLCAGISVYSPMKYHGLN---APGKHIGVLGLGGLGHVAVKFAKAFGMKVTVISS 171
Gan024020                      DKGAPLLCAGISVYSPMKYHGLN---APGKHIGVLGLGGLGHVAVKFAKAFGMKVTVISS 182
OsCAD7_Os04t0612700-01         DSGAPLLCAGVTVYAPMRQHGLC---EAGKHVGVVGLGGLGHVAVKFARAFGMRVTVIST 234
Ola022375.1                    DRGAPLLCAGVTVYAPMRQHGLS---EQGKHVGVLGLGGLGHVAVKFAKAFGMKVTVIST 221
Bam022082.1                    DSGAPLLCAGITVYAPMKHHGLC---EPGKHIGVLGLGGLGHVAVKFAKAFGMKVTVISR 220
PH02Gene32740.t1               DRGAPLLCAGITVYAPMKHHGLC---EPGKHIGVLGLGGLGHVAVKFAKAFGMKVTVIST 218
Bam044253.1                    DRGAPLLCAGITVYAPMKHHGLS---KPGKHIGVLGLGGLGHVAVKFAKAFGMKVTVLST 216
Rgu006342                      AGVAPLLCAGITVWEPLRAGGVG----EGTRVGVVGIGGLGHLAVRLAAALGAEVTAFTT 183
Rgu008067                      AAATPLLCAGITTYHPLKEHGAG----PGKRVGVIGMGGLGHVGVKIAAAMGADVAVLSR 285
Rgu021228                      AATTPLLCAGITTYAPLKRHGVG----SGSKVGVIGMGGLGHVAVKIAAAMGAEVSVLSR 217
Rgu020259                      AAAAPLLCAGITTYSPLRRWGAG----PGRRVAVVGLGGLGHMAVKIAHAMGAEVTVLSQ 207
Rgu021242                      DVAAPLLCAGITLYTPLTHWGAG----PGKKVAVVGMGGLGHVGVKIAHALGAEVTVLSQ 214
                                  :******:: :  :          .   .: *:**:** .::..  :*  * ..:
```

```
                              SXXK
Rgu021554                     GTAKADLA-RKLGAHHHVDT-ETTDAAAWLAERGGASVILATATSGEAISGLVGGLSGRG 254
Gan031993                     SESKRDEAISLLGADNFVIS---SDKQQMESLKGSLHYIVDTAAGDHPFDPYLSLLKVGG 284
OsCAD4_LOC_Os11g40690.1       SESKREEAINLLGADNFVIS---SDENQMESLKSSLHFIIDTASGDHQFDPYLSLLKVGG 258
Bam015552.1                   SESKRNEAINLLGADSFVIS---SDKEQMESLKSSLHFIIDTAAGDHPFDPYLSLLKVGG 276
OsCAD1.1_LOC_Os10g11810.1     SESKRKEAIDLLGADNFVVS---SDENQMETLKSSLNFIIDTASGDHPFDPYLTLLKVGG 269
OsCAD1.3_LOC_Os10g11810.3     SESKRKEAIDLLGADNFVVS---SDENQMETLKSSLNFIIDTASGDHPFDPYLTLLKVGG 212
Ola036924.1                   SESKRAEAISLLGAENFVIS---SDEKQMESLKSSLHFIVDTASGDHPMDPYLSLLKVGG 269
Rgu012272                     SESKRAEAISLLGADNFVIS---SDEKQMESLQSSLHFIVDTASGNHPMDPYLSLLKVGG 269
Bam025746.1                   SESKREEAISLLGADNFVIS---SDKKQMESLKSSLHFIVDTASGDHPFDPYLSLLKVGG 359
PH02Gene48685.t1              SESKREEAISLLGVDNFVIS---SDEKQMESLKSSLDFIVDTASGDHPFDPYLSLLKVGG 269
PH02Gene44682.t1              SESKREEAISLLGADNFVIS---SDEKQMESLKSSLDFIVDTASGDHPFDPYLSLLKVGG 269
PH02Gene46268.t1              SESKREEAISLLGADNFVIS---SDEKQMESLKSSLDFIVDTASGDHPFDPYLSLLKVGG 269
Ola035994.1                   SGKKRAEAMEHLGADAYLVS---SDADAMAAAADSLDYIIDTIPVHHPMEPYLALLKLDG 267
Rgu003339                     SGKKRAEAMEHLGADAYLVS---SDDAAMAAAADSLDYIIDTIPVHHPMEPYLALLKLDG 239
OsCAD2-1_Os02t0187800-01      SARKRGEAMDDLGADAYLVS---SDAAAMAAAGDSLDYIIDTVPVHHPLEPYLALLKLDG 269
OsCAD2-2_Os02t0187800-02      SARKRGEAMDDLGADAYLVS---SDAAAMAAAGDSLDYIIDTVPVHHPLEPYLALLKLDG 228
Gan017720                     SGKKRAEAMEHLGADAYLVS---SDADQLAAAADSLDYIIDTIPVHHPLEPYLALLKLDG 260
Bam010499.1                   SGKKHAEAIEHLGADAYLVS---SDADQMAAAVDSLDYIIDTIPVHHPLEPYLALLKLDG 269
PH02Gene39617.t1              SGKKRTEAMEHLGADAYLVS---SDADQMATAADSLDYIIDTIPVHHPLEPYLALLKMEG 269
PH02Gene04778.t1              SGKKRAEAMEHLGADAYLVS---SAAEQMAAAADSLDYIIDTIPVHHPLEPYLELLKLDG 269
Bam007854.1                   SGKKRTEAMEHLGADAYLVS---SDADQMAAAADSLDYIIDTIPVHHPLEPYLALLKLDG 269
OsCAD6_LOC_Os04g15920.1       SPAKEREARENLKADNFVVS---TDQKQMQAMTRSLDYIIDTVAATHSLGPILELLKVNG 273
Ola016985.1                   SPAKEREARESLKADDFVVS---TNEKQMQAMTRSLDYIIDTVSAKHSLGPILELLKVNG 254
Bam039144.1                   SPAKEREARESLKADDFVVS---TDEKQMQAMTRSLDYIIDTVSAKHSLGPILELLKVNG 254
Bam034885.1                   SSAKEREARESLKADDFVVS---TDEKQMQAMTRSLDYIIDTVSAKHSLGPILELLKVNG 270
Bam039795.1                   SPAKEREARESLKADDFVVS---TNGKQMQAMTRSLDYIIDTVSAKHSLGPILELLKVNG 270
PH02Gene48202.t1              SPAKEREARESLKADDFVVSRLSTNEKHMQAMKRSLDYIIDTVSAKHSLGPILELLKVNG 273
PH02Gene49901.t1              SPGKEREARERLKADDFVVS---TNEKQMQAMTRSLDYIIDTVSAKHSLGPILELLKVNG 270
Gan014390                     SPAKEREAREGLKADDFVVS---TNEKQMQAMTRSLDYIIDTVSAKHSLGPILELLKVNG 270
Rgu008819                     SAGKEAGAMEKLGADEFLLS---TDAEQMKAAIGTMDGIIDTVSAVHDLTPLVMLLRTHG 275
OsCAD9_LOC_Os03g12270.1       SPWKEAEAVERLGADAFLLS---TNAEQMKAAAGTMDGIIDTVSAVHDLTPLITLLRTHG 273
Gan006403                     SPRKEVEAVEKLGADEFLLS---TDAEQMKAAIGTMDGIIDTVSAVHDLTPLIMLLRTHG 199
Ola001888.1                   SASKEAEAVEKLGADEFLLS---TDAAQMKAAIGTMDGIIDTVSAVHDLTPLAMLLRTHG 266
PH02Gene26598.t1              SPGKEAEAVEKLGADEFLLN---TNTERMKAAIGTMDGIIDTVSAVHDLTPLIMLLKTHG 266
Bam045387.3                   SPGKEVEAVEKLGADEFVLS---SNAEQMKAATGTMDGIIDTVSAVHDLTPLIMLLRTHG 874
OsCAD8B_LOC_Os09g23540.1      SPGKRREALEHLGADEFLVS---RDAGQMAAAAGTMDGILNTVSAWHPVAPLFALMKPMA 342
OsCAD8C1_LOC_Os09g23550.1     SPGKRQEALEHLGADEFLVS---RDAGQMAAAAATMDGILNTVSAWHPIAPLFSLMKPMA 345
OsCAD8C2_LOC_Os09g23550.2     SPGKRQEALEHLGADEFLVS---RDAGQMAAAAATMDGILNTVSAWHPIAPLFSLMKPMA 211
Ola032369.1                   SPGKKQEALEHLGADEFLVS---RDPEQMKLAVGTMDGIIDTVSAWHPITPLFALMKPMG 268
Rgu014373                     SPGKKQEALEHLGADEFLVS---RDPEQMKGAVGTMDGIINTVSAWHPIAPLFALMKPMG 269
Gan002573                     SPGKRQEALEHLGADEFLVS---RDPEQMKAAVGTMDGIIDTVSAWHPLAPLFALMKPMG 243
Gan004029                     SPGKRQEALEHLGADEFLVS---RDPEQMKAAVGTMDGIINTVSAWHPIAPLFALMKPMG 241
PH02Gene13789.t1              SPGKRQEALEHLGADEFLVS---RDPEQMKAAVGTMDGIIDTVSAWHPIAPLFALMKPMG 268
Bam027739.1                   SPGKRQEALEHLGADEFLVS---RDPEQMKEAVGTMDGIIDTVSAWHPIAPLFALMKPMG 323
OsCAD8A_LOC_Os09g23530.1      SPAKRREEALERLGADAFLSS---RDGEGMAAAAATMDGIIDTVSAGHPLVPLLSLLKPKG 267
OsCAD8D_LOC_Os09g23560.1      SPAKRGEALGRLGADAFLSS---RDGEGMAAAAATMDGIIDTVSAGHPLVPLLSLLKPKG 267
Bam027740.1                   ------------------------------AAMSTMDGVIDTVSAGHPIVPLLALLKPMG 240
Bam040591.1                   SPGKRQEALEHLGADAFLVS---QDAEQMKAAMGTMDGIIDTVSAGHPIVPLLALLKPMG 266
PH02Gene13790.t1              SPGKRQEALEHLGADTFLVS---HDAEQMKAATGTMDGIIDTVSAGHPIVPLLALLKPMG 381
PH02Gene13791.t1              SPGKRQEALEHLGADTFLVS---HDAEQMKAATGTMDGIIDTVSAGHPIVPLLALLKPMG 267
OsCAD3_LOC_Os10g29470.1       SPGKKREALERLGADAFVVS---SSAEEMEAARSTMDGVINTVSANTPMAPYLALLKPNG 275
OsCAD5_LOC_Os08g16910.1       SPGKRQEALERLGADAFVVS---KNADEMNAATGTMDGIINTVSANIPIAPLLGLLKPNG 265
Rgu015472                     SPGKKQEALERLGADAFVVS---SNPDEMKGATGTMDGIINTVSANMPMAPLLALLKPNG 288
Ola032840.1                   SPGKKQEALERLGADAFVVS---SNADDMKSATGTMDGIINTVSASIPMAPLLGLLKPNG 276
PH02Gene38471.t1              SPGKKQEALERLGADAFVVS---SNAEEMKAAMGTIDGIINTVSANIPVAPLRPAE---- 271
Bam005700.1                   SPGKKQEALERLGADAFVIS---SNAEEMKAAMGTMDGIINTVSANIPVAPLFGLLKSNG 276
Bam017920.1                   SPGKKQEALERLGADAFVIS---SNAEEMKAAMGTMDGIINTVSANIPVAPLFGLLKSNG 276
PH02Gene38470.t1              SPGKKQEALERLGADAFVVS---SNAEEMKAAMGTMDGIINTVSANIPMAPLFGLLKPNG 273
Bam032152.1                   SPGKKQEALERLGADAFVVS---SNAEEMKTAMGTMDGIINTVSANIPMVPLFGLLKPNG 276
Gan021750                     SPGKKQEALERLGADAFVVS---SNAEEMKAATGTMDGIINTVSANIAVAPLFVLLKPNG 228
Gan024020                     SPGKKQEALERLGADAFVVS---SNAEEMKAATGTMDGIINTVSANIAVAPLFVLLKPNG 239
OsCAD7_Os04t0612700-01        SPVKRQEALERLGADGFIVS---TNASEMKAAMGTMHGIINTASASTSMHSYLALLKPKG 291
Ola022375.1                   SPEKRQDALEKLGADGFVLS---SNASEMKSMAGTMHGIINTACASTSMYPYFALLKPRG 278
Bam022082.1                   SPEKRQEALEKLGADDFVVS---SNANEMKAVMGTMHGILNTACASTSMYAYFGLLKPHG 277
PH02Gene32740.t1              SPEKRQEALEKLGADAFVVS---SNANEMKAVMGTMHGIINTACASTSMYPYFGLLKPHG 275
Bam044253.1                   SPEKRQEALEKLGADAFVVS---SNANEMKAVMGTMHGIINTACASTSMYPYFGLLKPHG 273
Rgu006342                     SAAKADDA-RRLGATRVVVS---TDADQMASVADSLDVVVDTVSVEHDLAPYLACVDTDG 239
Rgu008067                     TDAKAEDG-KAFGAAEYFAT---EDESVFTEQAGSFDILLNTVGSGIDLDAFMGLLGRGG 341
Rgu021228                     TAAKEEDG-KKFGAAHYYAT---EDESVFEDLAGHYDLLLNTVGDGIDLDAFLGTLGRGG 273
Rgu020259                     SLKKQEDG-LRLGADRYHAT---SDPDTFEQLAGSFDLIVNTVSAHIDIAAYLGLLAVDG 263
Rgu021242                     TLSKEADG-KRLGADHYYAT---SDEATFRELDSSFDLIVNTVSADLELERYVALLKLDG 270
                                                                    :: *      .
```

```
Rgu021554                    QLLVVGAAESPLTLDAMSLIGPVASVAGHA----------------------SGTSKDSE 292
Gan031993                    VMALVGFPSEI-KLHPANLNRGARTLAGSI----------------------TGGTKGIQ 321
OsCAD4_LOC_Os11g40690.1      VMVLLSFPSEI-KVHPENLNLAARSLAGSV----------------------TGGTKDIQ 295
Bam015552.1                  VMALVGFPSEI-KVHPANLVLGAQTLSGSL----------------------TGGTKDTQ 313
OsCAD1.1_LOC_Os10g11810.1    VMALLSFPSEI-KVHPANLNLGGRSLSGSV----------------------TGGTKDIQ 306
OsCAD1.3_LOC_Os10g11810.3    VMALLSFPSEI-KVHPANLNLGGRSLSGSV----------------------TGGTKDIQ 249
Ola036924.1                  VMVLLSFPEEI-KVHPANLNLGARSLSGSV----------------------TGGTKDTQ 306
Rgu012272                    VMTLLSFPEEI-KVHPGNLNLGARSLSGSV----------------------TGGTKDTQ 306
Bam025746.1                  VMALVGFPKEI-RVHPANLNLGARSLAGSV----------------------TGGTKDTQ 396
PH02Gene48685.t1             VMTLLSFPKEI-KVHPANLNLGARSLSGSV----------------------TGGTKDTQ 306
PH02Gene44682.t1             VMVLLSFPKEI-KVDPRNLNLGARSLSGSV----------------------TGGTKDTQ 306
PH02Gene46268.t1             VMALLSFPKEI-KVHPANLNLGARTLSGSV----------------------TGGTKDTQ 306
Ola035994.1                  KHILLGVIGQPLSFVSPMVMLGRKSITGSF----------------------IGSVDETA 305
Rgu003339                    KHVLLGVIGQPLSFVSPMVMLGRKSITGSF----------------------IGSVDETA 277
OsCAD2-1_Os02t0187800-01     KLILMGVINQPLSFISPMVMLGRKAITGSF----------------------IGSMAETE 307
OsCAD2-2_Os02t0187800-02     KLILMGVINQPLSFISPMVMLGRKAITGSF----------------------IGSMAETE 266
Gan017720                    KHILMGVIGQPLSFVTPMVMLGRKSITGSF----------------------IGSMDETA 298
Bam010499.1                  KLILMGVIGQPLSFVTPMVMLGRKSITGSF----------------------IGSIDETE 307
PH02Gene39617.t1             KLILMGVIGQPLSFVTPMVMLGRKSITGSF----------------------IGSMDETE 307
PH02Gene04778.t1             KHILMGVIGQPLSFVTPMVMLGRKSITGSF----------------------IGSMDETE 307
Bam007854.1                  KHILMGVIGQPLSFVTPMVMLGRKSITGSF----------------------IGSMDETE 307
OsCAD6_LOC_Os04g15920.1      KLVLVGAPEKPVELPSFPLIFGKRTVSGSM----------------------TGGMKETQ 311
Ola016985.1                  KLVLVGAPDKPVELPSFPLIFGKRTVSGSM----------------------TGGMRETQ 292
Bam039144.1                  KLVLVGAPDKPVELPSFPLIFGKRTVSGSM----------------------TGGMKETQ 292
Bam034885.1                  KLVLVGAPDKPVELPSFPLIFGKRTVSGSM----------------------TGGMKETQ 308
Bam039795.1                  KLVLVAAPDKPVELPSFPLIFGKRTVSGSM----------------------TGGMEETQ 308
PH02Gene48202.t1             KLVLVAAPDKPVELPSFPLIFGKRTVSGSM----------------------TGGMKETQ 311
PH02Gene49901.t1             KLVLVAAPDKPVELPSFPLIFGKRTVSGSM----------------------TGGMKETQ 308
Gan014390                    KLVLVGAPDKPVELPSFPLIFGKRTVSGNM----------------------TGGMKETQ 308
Rgu008819                    KLVPVGAPGKPVQLALYPLQSGGKSVAGSM----------------------IGGMKETQ 313
OsCAD9_LOC_Os03g12270.1      QLVPVGSPGKPVQLALYPLQSDGKSVAGSM----------------------IGGMRDTQ 311
Gan006403                    KLVPVGSPGKPVQLALYPLQSGGKTVAGSMIGGMGDTQEMIHLGGKTVAGSMIGGMGDTQ 259
Ola001888.1                  KLVPVGSPGKPVQLALYPLQSGGKMVAGSM----------------------IGGMKETQ 304
PH02Gene26598.t1             KLVPVGSPGKPVQLALYPLQSGGKIVAGSM----------------------IGGMRDTQ 304
Bam045387.3                  KLVPVGSPGEPVQLALYPLQSGGKVVAGSM----------------------IGGMGDTQ 912
OsCAD8B_LOC_Os09g23540.1     QMVFVGAPTRPLELPAYAIVPGGKGITGNC----------------------VGGIRDCQ 380
OsCAD8C1_LOC_Os09g23550.1    QMVFVGGPTRPLELPAYAIVPGGKGITGNC----------------------VGGIRDCQ 383
OsCAD8C2_LOC_Os09g23550.2    QMVFVGGPTRPLELPAYAIVPGGKGITGNC----------------------VGGIRDCQ 249
Ola032369.1                  QMVFVGGPTKPLELPAYAIVPGGKGIAGNC----------------------VGGVKHCQ 306
Rgu014373                    QMVFVGGPTRPLELPAYAIVPGGKGIAGNC----------------------VGGVKDCQ 307
Gan002573                    QMVLVGGPTKPLELPAYAVVPGGKGIAGNC----------------------VGGIRDCQ 281
Gan004029                    QMVFVGGPTKPLELPAYAIVPGGKGIAGNC----------------------VGGIRDCQ 279
PH02Gene13789.t1             QMVFVGGPTKPLELPAYAIVPGGKGIAGNC----------------------VGGIRDCQ 306
Bam027739.1                  QIVFVGGPTKPLELPAYAIVPGGKGIAGNC----------------------VGGMRDCQ 361
OsCAD8A_LOC_Os09g23530.1     QMVVVGAPAAPLQLPAIAIIDGGKRVAGSG----------------------GGSVAECQ 305
OsCAD8D_LOC_Os09g23560.1     QMVVVGAPAMPLQLPAYAIIEGGKRVAGNG----------------------VGSVAECQ 305
Bam027740.1                  QMVVVGVPSKPLELPAYAITTGGKRVAGNG----------------------VGGVADCQ 278
Bam040591.1                  QMVVVGAPSKPLELPAYAIITGGKRVAGNG----------------------VGSVGDCQ 304
PH02Gene13790.t1             QMVVVGAPNKPLELPAYAIITGGKRVAGSG----------------------VGGVGDCQ 419
PH02Gene13791.t1             QMVVVGAPNKPLELPAYAIITGGKRVAGSG----------------------VGSVGDCQ 305
OsCAD3_LOC_Os10g29470.1      KMILVGLPENPLEVPPFSLVHGNRTLAGSN----------------------IGGMADTQ 313
OsCAD5_LOC_Os08g16910.1      KMILVGLPEKPMEIPPFALVA--------------------------------------- 286
Rgu015472                    KMILLGLPDNPLEVPAFCLVAASRTLAGSC----------------------IGGMRDTQ 326
Ola032840.1                  KMILVGLPENPLEVPAFSLVAANKTLAGSC----------------------IGGMRDTQ 314
PH02Gene38471.t1             --------------AQWQDDPANKTLAGSC----------------------IGGMRDTQ 295
Bam005700.1                  KMILVGLPEKPLEVPAFALVQMNRTLCGSC----------------------IGGMRDTQ 314
Bam017920.1                  KMILVGLPEKPLEVPAFALVQSKFIRVT-------------------------------- 304
PH02Gene38470.t1             KMILVGLPDKPLEVPAFALVATNKTLAGSC----------------------IGGMRDTQ 311
Bam032152.1                  KMILVGLPEKPLEVPAFALVQTNKTLAGSC----------------------IGGMRDTQ 314
Gan021750                    KMILVGLPEKPLEVPAFALVQMNKTLAGSC----------------------IGGMRDTQ 266
Gan024020                    KMILVGLPEKPLEIPAFALVQMNKTLAGSC----------------------IGGMRDTQ 277
OsCAD7_Os04t0612700-01       KMILVGLPEKPLQIPTFALVGGGKILAGSC----------------------MGSISETQ 329
Ola022375.1                  KIILVGLPEKPLQLPAFALVGGGKILAGSC----------------------MGSIEETQ 316
Bam022082.1                  KMILVGLPEKPLQVPAFALVGGGKALAGSC----------------------MGSIRDTQ 315
PH02Gene32740.t1             KMILVGLPEKPLQVPAFALVGGGKTLAGSC----------------------MGSIKDTQ 313
Bam044253.1                  KMILVGLPEKPLQVSAFALVGGGKTLAGSC----------------------MGSIKDTQ 311
Rgu006342                    VLYPVGYL-GPLSVDSMSLLPGRKRLSSSG----------------------SGGVDRTA 276
Rgu008067                    VMANLGAPSDNLEVSAFSLLPNRRSIAGSM----------------------VGGLPETQ 379
Rgu021228                    VMVNLGAPSDKLEVGAFSLLPFRRSIEGSM----------------------VGGLPETQ 311
Rgu020259                    ALVNVGAPAEPFTVHAMTLVGGRRTFAGSM----------------------IGGIAETQ 301
Rgu021242                    AMVNVGIPATPGSIHAFSLANGRRSLSGSK----------------------IGGIRDTQ 308
```

```
Rgu021554                   DTLSFCRLTAVRPMVETMPLERAGEAYERMMANDARFRMVLTTGQ--------------- 337
Gan031993                   EMLNLCAAKKIYPEIEIIKMDYINEALKRLVNRDVKYRFVIDIENSFNQYN--------- 372
OsCAD4_LOC_Os11g40690.1     EMINFCAANNVYPDIEMIKIDYVNEALQRLINRDVRFRFVIDIENSFK*----------- 343
Bam015552.1                 EMLNFCAANKIYPEIEIIKMDNINEALERLINRDVKYRFVGGVMALVGFPSEIKVHPANL 373
OsCAD1.1_LOC_Os10g11810.1   EMINFCAANKIYPDIEMIKIDYINEALQRLVDRDVRFRFVIDIENSFK*----------- 354
OsCAD1.3_LOC_Os10g11810.3   EMINFCAANKIYPDIEMIKIDYINEALQRLVDRDVRFRFVIDIENSFK*----------- 297
Ola036924.1                 EMINFCAANKIYPEIEIVKMDYINEALERLIKRDVRYRFVIDIENSFK------------ 354
Rgu012272                   EMINFCAANKIYPDIEMINIDYINEALDRLIKKDVRYRFVIDMENSFK------------ 354
Bam025746.1                 EMINFCAANKIYPDIEMIKIDYINEALERLINRDVRYRFVIDIETSFK------------ 444
PH02Gene48685.t1            EMINFCAANKIYPDIEMIKIDYINEALERLINRDVRYRFVIDIENSFK------------ 354
PH02Gene44682.t1            EMINFCAANKIYPDIEIIKIDYINEALERLINRDARYRFVIDIENSFK------------ 354
PH02Gene46268.t1            QMLNFCAANKIYPDIEMIKIDYINEALERLINRDVRYRFVIDIENSFK------------ 354
Ola035994.1                 EVLQFCVDKGLTSEIEVVKMGYVNQALERLERNDVRYRFVVDVAGSNLEDDAATDLA--- 362
Rgu003339                   EVLQFCVDKGLTSQIEVVKMDYVNQALKRLERNDVRYRFVVDVAGSNLEASAADLP---- 333
OsCAD2-1_Os02t0187800-01    EVLNFCVDKGLTSQIEVVKMDYVNQALERLERNDVRYRFVVDVAGSNIDDADAPPA*--- 363
OsCAD2-2_Os02t0187800-02    EVLNFCVDKGLTSQIEVVKMDYVNQALERLERNDVRYRFVVDVAGSNIDDADAPPA*--- 322
Gan017720                   EVLQFCVDKGLTSQIEVVKMDYVNQAIERLERNDVRYRFVVDVAGSNIEDAA-------- 350
Bam010499.1                 EVLQFCVDKGLTSQIEVVKMDYVNQALERLERNDVRYRFVVDVAGSNIVDAA-------- 359
PH02Gene39617.t1            EVLQFCVDKGLTSQIEVVKMDYVNQALERLERNDVRYRFVVDVAGSNIEDAA-------- 359
PH02Gene04778.t1            EVLQFCVDKGLTSQIEVVKMDYVNQAFERLERNDVRYRFVVDVAGSNIEDAA-------- 359
Bam007854.1                 EVLQFCVDKGLTSQIEVVKMDYVNQALERLERNDVRYRFVLDVAGSNIEDAA-------- 359
OsCAD6_LOC_Os04g15920.1     EMMDICGEHNITCDIEIVSTDRINDALARLARNDVRYRFVINVGGDSKL*---------- 360
Ola016985.1                 EMMDLCGQHNITCDIELVSPDRINEALGRLARNDVRYRFVVDIESSSKL----------- 341
Bam039144.1                 EMMDLCGEHDITCDIELVSTDRINEALGRLARNDVRYRFVIDIGKNSKL----------- 341
Bam034885.1                 EMMDLCGEHDITCDIELVSTDRINEALGRLARNDVRYRFVIDIGKNSKL----------- 357
Bam039795.1                 EMMDMCGEHNITCDIELVSTDRINEALGRLARNDVRYRFVIDIGKNPKL----------- 357
PH02Gene48202.t1            EMMDLCGEHNITCDIELVSTDRINEALGRLARNDVRYRFVVDIGKNSKLQ---------- 361
PH02Gene49901.t1            EMMDLCGEHNITCDIELVSTDRINEALGRLARNDVRYRFVVDIGKNSKL----------- 357
Gan014390                   EMMDLCGEHNITCDIELVSTDRINEALGRLARNDVRYRFVVDIAKNSKL----------- 357
Rgu008819                   EMIDFAAEHNITAQVEVIGIEDVNAAMERLQKGDVRYRFVIDIANTIARA---------- 363
OsCAD9_LOC_Os03g12270.1     EMVDFAVEHGVAAEVEVIGMEDVNGAMERLQKGDVRYRFVIDVANTMARAR*-------- 362
Gan006403                   EMIDFAAEHNIAAEVEVIGIEDVNAAMERLQRGDVRYRFVIDVANTMARAY--------- 310
Ola001888.1                 EMIDFAAEHNITAQVEVIGMEDVNAAMERLQKGDVRYSSTAIKGEKKGWR---------- 354
PH02Gene26598.t1            EMIDFAAEHSIAAEVEVIGIGDVNAAMERLQKGDVRYRFVIDIANTMARA---------- 354
Bam045387.3                 EMIDFAAEHNIAAEVEVIGIGDVNAAMERLQRGDVRYRFVIDVANTMARAY--------- 963
OsCAD8B_LOC_Os09g23540.1    AMLDFAGEHGITAEVEVIKMDYVNTAMERLEKNDVRYRFVIDVAGSSLGGSGDDKI*--- 436
OsCAD8C1_LOC_Os09g23550.1   AMLDFAGEHGITAEVEVIKMDYVNTAMERLEKNDVRYRFVIDVAGSSLAGSGDAKI*--- 439
OsCAD8C2_LOC_Os09g23550.2   AMLDFAGEHGITAEVEVIKMDYVNTAMERLEKNDVRYRFVIDVAGSSLAGSGDAKI*--- 305
Ola032369.1                 AMLDFAAAHGITAEVEVVKMDYVNTALERLEKNDVRYRFVIDVAGSLGSTA--------- 357
Rgu014373                   AMLDFAAEHGITAEVEVIKMDYVNTALERLEKNDVRYRFVVDVAGSLGSDA--------- 358
Gan002573                   AMLDFAGEHGVTADVEVIKMDYANKALERLEKNDVRFRFVIDVAGSLGSTS--------- 332
Gan004029                   AMLDFAGEHGITAEVEVIKMDYVNTALERLEKNDVRYRFVIDIAGSLGSAA--------- 330
PH02Gene13789.t1            AMLNFAGEHGITAEVEVIKMDYVNTALERLEKNDVRYRFVIDVAGSLGSTA--------- 357
Bam027739.1                 AMLDFAGEHGITAEVEVIKMDYVNTALERLEKNDVRYRFVIDVAGSLGSTA--------- 412
OsCAD8A_LOC_Os09g23530.1    AMLDFAGEHGIAADVEVVAMGDVNAALGRLERNDVRYRFVIDVAGTLHAAAPS*------ 359
OsCAD8D_LOC_Os09g23560.1    AMLDFAGEHGIAADVEVVAMDAVNAALGRLERNDVRYRFVVDVAGTMHAAAAAAASS*-- 362
Bam027740.1                 AMLDFAGEHGITADVEVVKMDYVNTAVERLERNDVRYRFVVDVAGSLGAAA--------- 329
Bam040591.1                 AMLDFAGQHGITADVEIVKMDYVNTAVERLERNDVRYRFVVDVAGSLGAAA--------- 355
PH02Gene13790.t1            AMLDFAGENGITADVEVVKMDYVNTAVERLERNDVRYRFVLDVAGSLGAAA--------- 470
PH02Gene13791.t1            AMLDFAGKHGITADVEVVKMDYVNTAVERLERNDVRYRFVLDVAGSLGAAA--------- 356
OsCAD3_LOC_Os10g29470.1     EMIELAAKHGVTADIEVIGADDVNTAMERLAKADVRYRFVIDVGNTLHAAAAE*------ 366
OsCAD5_LOC_Os08g16910.1     ------SKHGVTAEIEVIGADYVNTAMERLAKADVRYRFVIDIGNTLKDAIE*------- 332
Rgu015472                   EMMDLAAEHGVTADVEVISADYVNAAMERLAEADVRYRFVIDVGNTLKSTV--------- 377
Ola032840.1                 EMIDLAAKHGVTADVEVISADYVNTAMERLAKADVRYRFVIDVGNTLTNTE--------- 365
PH02Gene38471.t1            EMIDLAAKHGVTADIEVISANYVNTAMERLAKADVRRLRIL------------------- 336
Bam005700.1                 EMIDLAAKHGVTADIEVIGADYVNTAMERLARADVRSTGDDDVVIKILYCGICHSDLHSI 374
Bam017920.1                 ------------------------------------------------------------ 304
PH02Gene38470.t1            EMIDLAAKHGVTADIEVISANYVNTAMERLAKADVRYRFVIDIGNTLENTE--------- 362
Bam032152.1                 EMIDLAAKHGVTADIEVIGAEYVNTALERLAKADVRYRFVIDIGNTLKNTE--------- 365
Gan021750                   EMINLAAKHGVTADVEVIGADYVNTAMERLAKADVRYRFVIDIGNTLKDTE--------- 317
Gan024020                   EMIDLAAKHGVTADVEVIGADYVNTAMERLAKADVRYRFVIDIGNTLKDTE--------- 328
OsCAD7_Os04t0612700-01      EMIDFAAEHGVAADIELIGADEVNTAMERLAKGDVRYRFVVDIGNTLRSD*--------- 379
Ola022375.1                 EMIDFAAEHGVAADIELVGAGGVNAAMERLAKGDVRYRFVIDVGDTLKSSD--------- 367
Bam022082.1                 EMIDFAAKHGVTADIELIGADGVNTAMERLAKGDIRYRFVIDVGNTLKSD---------- 365
PH02Gene32740.t1            EMIDFAAKHGVTADIELVGADCVNTAMERLAKGDVRYRFVIDVGNTLKSD---------- 363
Bam044253.1                 EMIDFAAKHGVTADIELISADSVNTAMERLAKGDIRYRFVIDVGNTLKSD---------- 361
Rgu006342                   ELLAFCGEHGITADVEVLPASEVATALDRLARNDVRYRFVLDTTAL-------------- 322
Rgu008067                   EMMDFCAEHGITATVEVITADQVDEYYDKVVSGDVRYRAVIDTSSLA------------- 426
Rgu021228                   EMLDFCAEHGITATVEVIDADDVDAYYDKVVSGDVRYRAVIDTATFG------------- 358
Rgu020259                   EMLDFCAEHGIGSDVEVIGAEQVNEAYERVLASDVRYRFVIDTASLA------------- 348
Rgu021242                   KMLDFCAEHHIGAEIETIGVDDVNKAYDRVVDSDVRYRFVIDTATFGG------------ 356
```
