# Supplementary material for: Homo- and Hetero-Dimers of CAD Enzymes Regulate Lignification and Abiotic Stress Response in Moso Bamboo
Source: Int J Mol Sci. 2021 Nov 29;22(23):12917. doi: 10.3390/ijms222312917 (PMC8657895; doi:10.3390/ijms222312917)

File S2: Uncropped and cropped western blot figures

- Uncropped image of Input total proteins detected with anti-HA

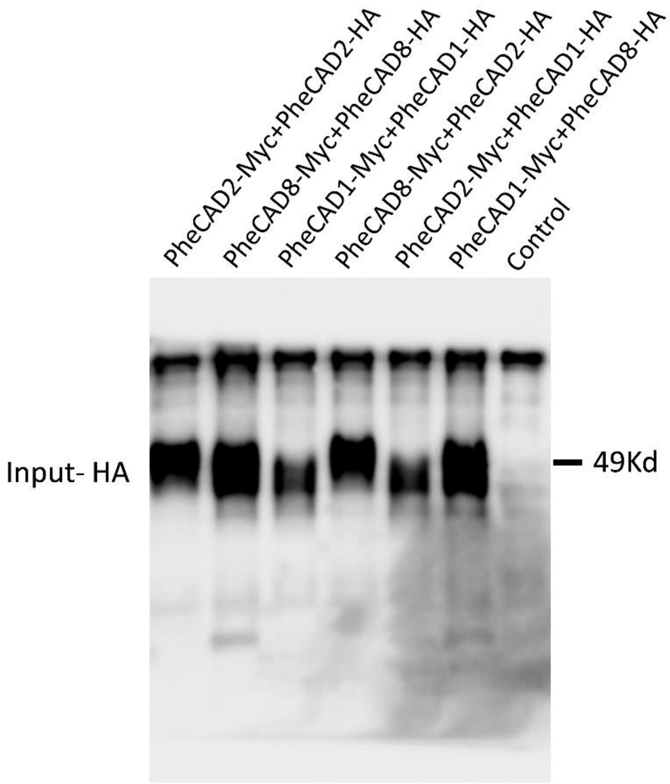

- Cropped image of Input total proteins detected with anti-HA

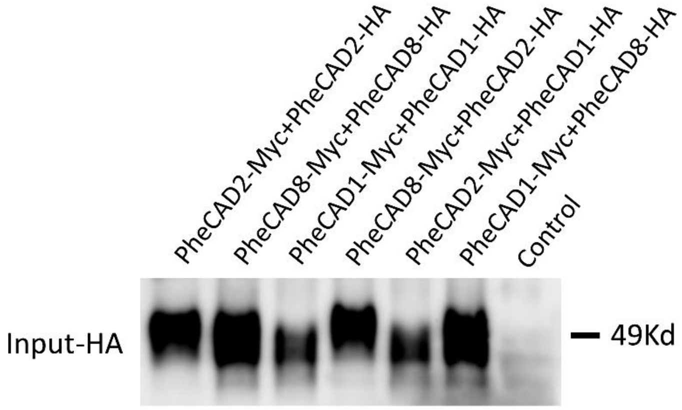

- Uncropped figure of Input total proteins detected with anti-Myc

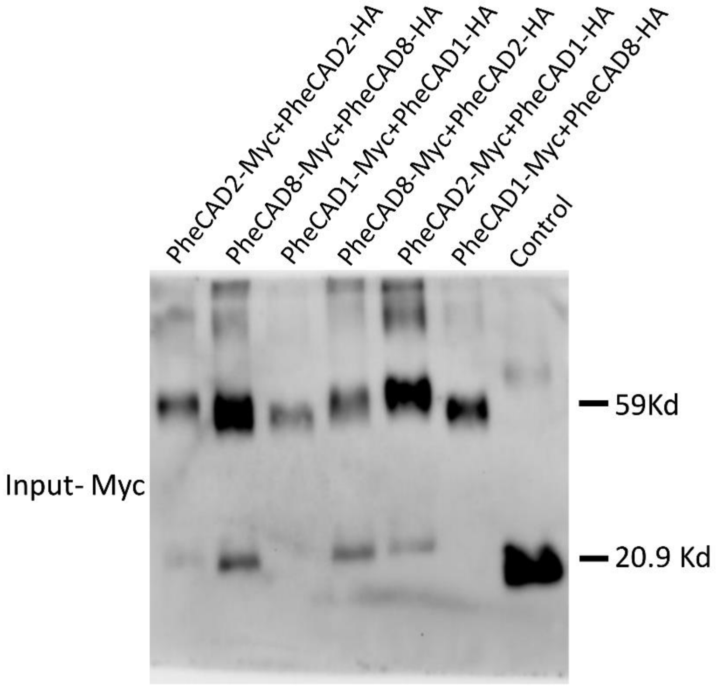

- Cropped figure of Input total proteins detected with anti-Myc

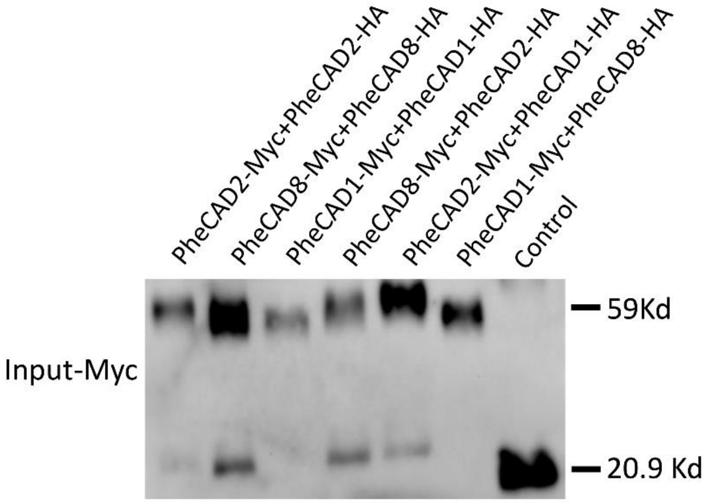

- Uncropped figure of IP proteins detected with anti-HA

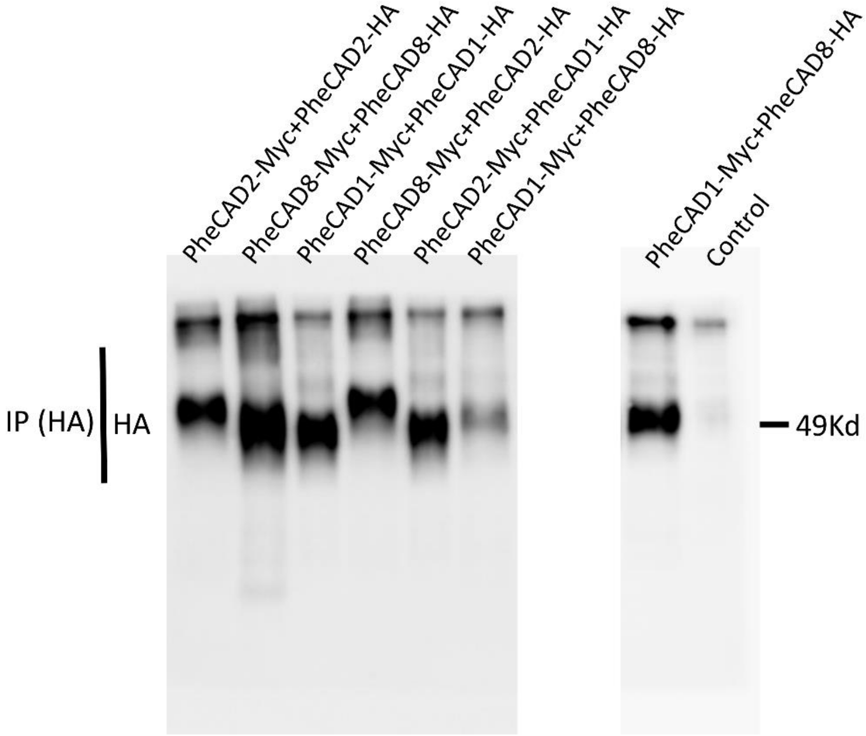

- Cropped figure of IP proteins detected with anti-HA

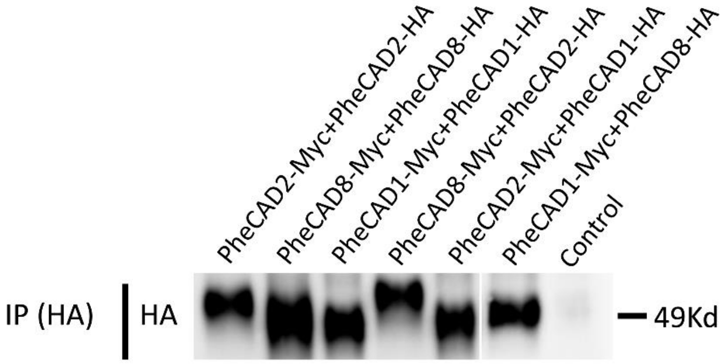

- Uncropped figure of IP proteins detected with anti-Myc

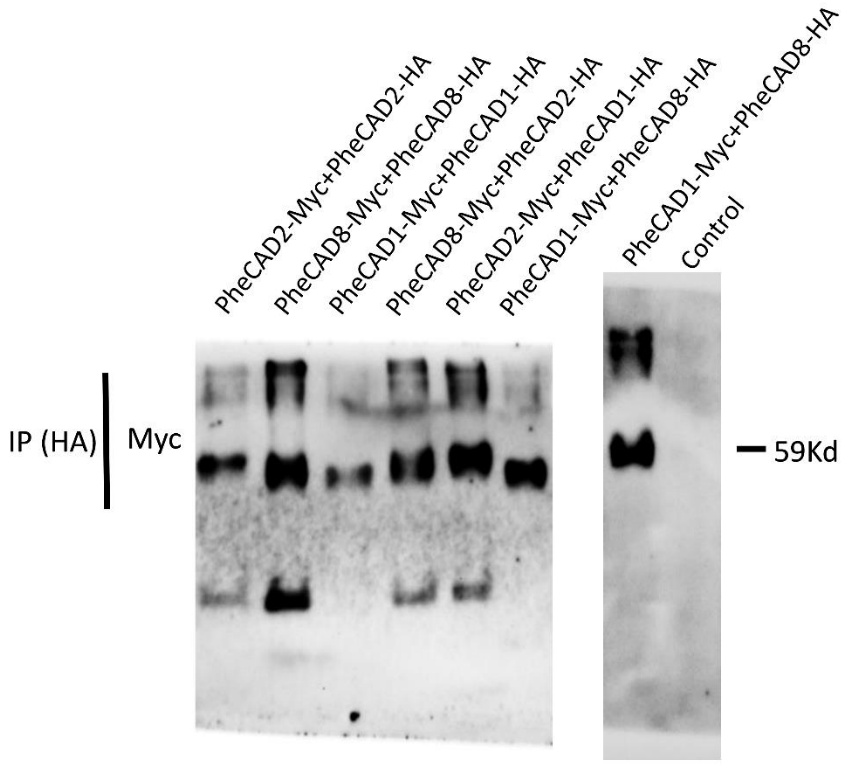

- Cropped figure of IP proteins detected with anti-Myc

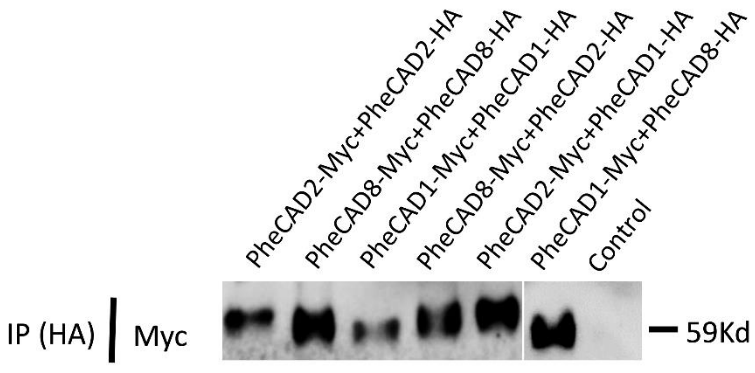

Supplement: Supplementary file 1 [file ijms-22-12917-s001.zip › Supplementary files/File S2.pdf]
