# Supplementary figures and images for: Homo- and Hetero-Dimers of CAD Enzymes Regulate Lignification and Abiotic Stress Response in Moso Bamboo
Source: Int J Mol Sci. 2021 Nov 29;22(23):12917. doi: 10.3390/ijms222312917 (PMC8657895; doi:10.3390/ijms222312917)

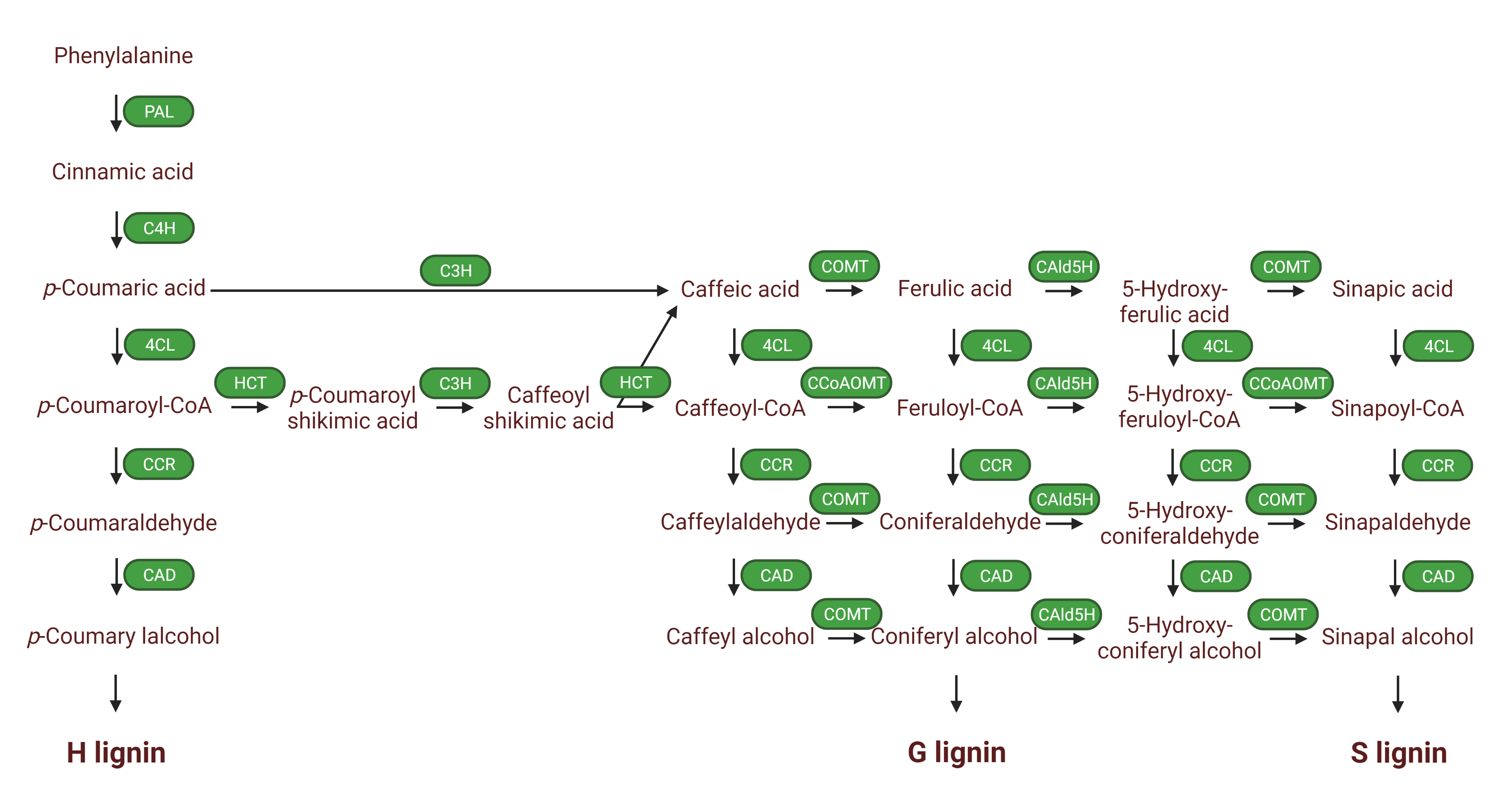

Supplement: Supplementary file 1 [file ijms-22-12917-s001.zip › Supplementary files/Figure S1.png]

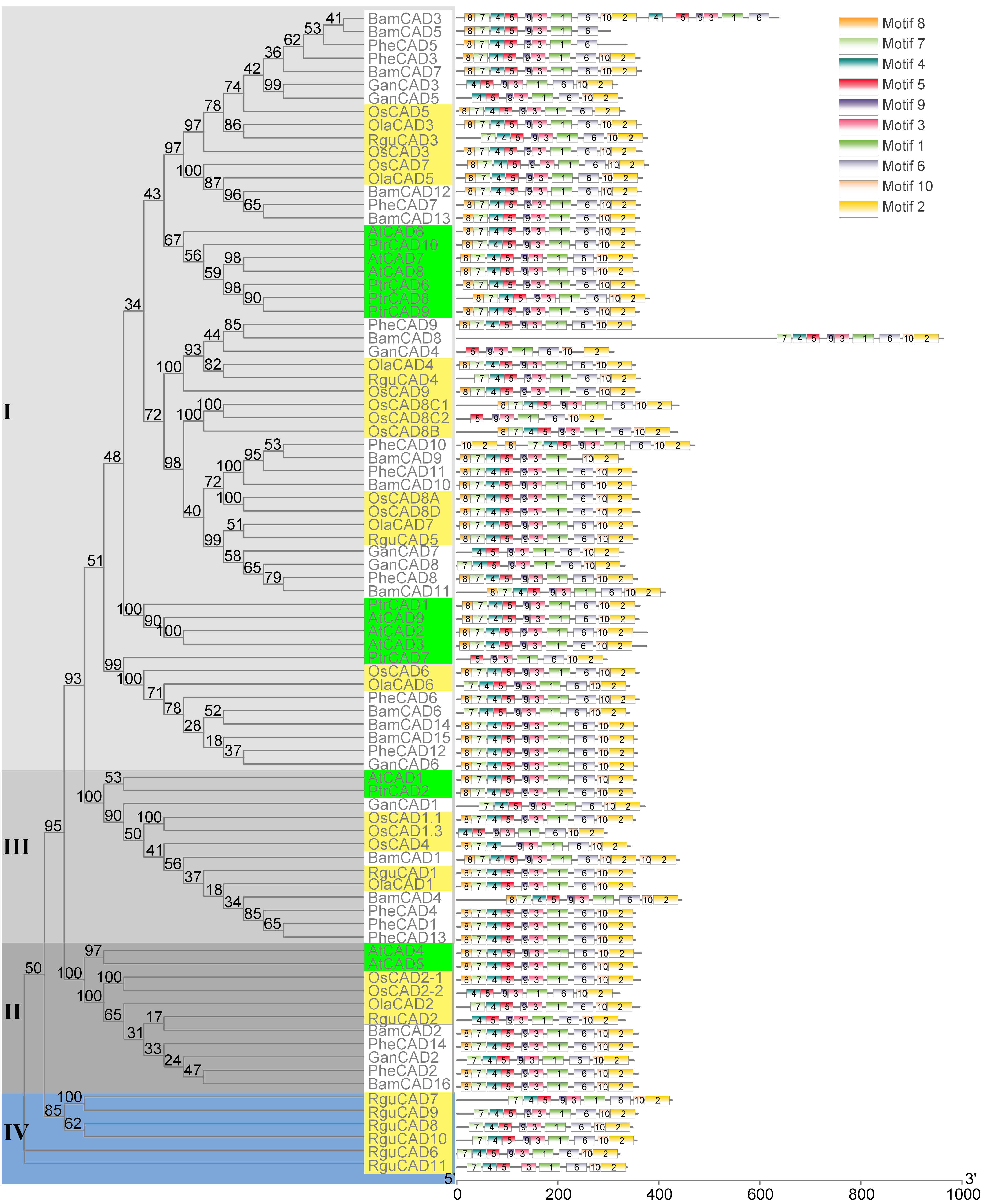

Supplement: Supplementary file 1 [file ijms-22-12917-s001.zip › Supplementary files/Figure S2.tif]
